# Functional Connectivity Basis and Underlying Cognitive Mechanisms for Gender Differences in Guilt Aversion

**DOI:** 10.1523/ENEURO.0226-21.2021

**Published:** 2021-12-15

**Authors:** Tsuyoshi Nihonsugi, Shotaro Numano, Masahiko Haruno

**Affiliations:** 1 Faculty of Economics, Osaka University of Economics, Osaka 533-8533, Japan; 2 Center for Information and Neural Networks, National Institute of Information and Communications Technology, Osaka 565-0871, Japan; 3 Graduate School of Frontier Biosciences, Osaka University, Osaka 565-0871, Japan

**Keywords:** DLPFC, fMRI, gender difference, guilt aversion, prosocial behavior, social norm

## Abstract

Prosocial behavior is pivotal to our society. Guilt aversion, which describes the tendency to reduce the discrepancy between a partner’s expectation and his/her actual outcome, drives human prosocial behavior as does well-known inequity aversion. Although women are reported to be more inequity averse than men, gender differences in guilt aversion remain unexplored. Here, we conducted a functional magnetic resonance imaging (fMRI) study (*n *=* *52) and a large-scale online behavioral study (*n *=* *4723) of a trust game designed to investigate guilt and inequity aversions. The fMRI study demonstrated that men exhibited stronger guilt aversion and recruited right dorsolateral prefrontal cortex (DLPFC)-ventromedial PFC (VMPFC) connectivity more for guilt aversion than women, while VMPFC-dorsal medial PFC (DMPFC) connectivity was commonly used in both genders. Furthermore, our regression analysis of the online behavioral data collected with Big Five and demographic factors replicated the gender differences and revealed that Big Five Conscientiousness (rule-based decision) correlated with guilt aversion only in men, but Agreeableness (empathetic consideration) correlated with guilt aversion in both genders. Thus, this study suggests that gender differences in prosocial behavior are heterogeneous depending on underlying motives in the brain and that the consideration of social norms plays a key role in the stronger guilt aversion in men.

## Significance Statement

Although women are reported to be more prosocial than men in terms of inequity aversion, gender differences in prosocial behavior based on guilt aversion are far less explored. Here, we conducted a functional magnetic resonance imaging (fMRI) study and a large-scale online behavioral study to address gender differences in guilt aversion. We demonstrate that men are more sensitive to guilt aversion than women, and a prefrontal social-norm network is key to men’s predominance in guilt-based prosocial behavior. These findings revealed the heterogeneity of gender differences in prosocial behavior depending on underlying motives and underlying neural mechanisms.

## Introduction

Prosocial behaviors are fundamental to human society. The most perceived motivation behind prosocial behaviors is inequity aversion (Fehr and Schmidt, 1999), which is defined as the propensity to avoid an imbalance between outcomes for the self and the other person. A great deal of behavioral research (Bolton and Katok, 1995; Eckel and Grossman, 1998; Andreoni and Vesterlund, 2001; Dickinson and Tiefenthaler, 2002; Croson and Gneezy, 2009; Kamas and Preston, 2015; Grosch and Rau, 2017) has accumulated evidence that women are more prosocial than men, since women are more inequity-averse.

However, economic research has shown that human prosocial behavior depends on not only preferred behavioral outcomes (e.g., fairness), but also on the belief of others (for review, see Fehr and Schmidt, 2006). People tend to live up to the expectations of others, since they suffer from guilt if they disappoint others (Baumeister et al., 1994). In behavioral game theory, this psychological process is named “guilt aversion” (Charness and Dufwenberg, 2006; Battigalli and Dufwenberg, 2007, 2009), in which an individual dislikes disappointing another person relative to what the other person believes they should receive (see Materials and Methods for a more detailed definition). However, gender differences in guilt aversion have been far less explored.

Previous functional magnetic resonance imaging (fMRI) studies of guilt aversion have revealed involvement of the dorsolateral prefrontal cortex (DLPFC), dorsal medial PFC (DMPFC), ventromedial PFC (VMPFC), insula, supplementary motor area, and temporal parietal junction (Chang et al., 2011; Nihonsugi et al., 2015; van Baar et al., 2019). For instance, it was demonstrated that the DLPFC is causally involved in the implementation of guilt aversion by integrating fMRI and transcranial direct current stimulation (tDCS; Nihonsugi et al., 2015). Considering these contributions of prefrontal cortices in guilt aversion, we assumed that prefrontal network interactions among the DLPFC, DMPFC, and VMPFC play a key role in producing the gender difference in guilt aversion, if any. In particular, the VMPFC may well be central to the gender difference in guilt aversion because several lesion studies (Tranel et al., 2005; Sutterer et al., 2015) demonstrated that the VMPFC is involved in the gender differences in social cognition.

Additionally, it is also possible that the gender difference in guilt aversion may reflect different cognitive strategies used by men and women. Guilt aversion requires the ability to assess another individual’s expectations and directly relates to his or her disappointment (i.e., empathy or theory of mind; Hoffman, 1982). At the same time, guilt aversion is a normative behavior elicited by experience (i.e., rule-based decisions; Haidt, 2003). Therefore, we also hypothesized that if there is a gender difference in guilt aversion, these two potential cognitive strategies: empathetic consideration and rule-based decision-making may contribute to the difference.

Regarding inequity aversion, previous fMRI studies (Haruno and Frith, 2010; Tricomi et al., 2010; Gospic et al., 2011; Crockett et al., 2013; Haruno et al., 2014; Tanaka et al., 2017) revealed involvement of the ventral striatum and amygdala. An integration of pharmacological intervention and fMRI also demonstrated that activity in the ventral striatum is critical for gender differences in this aversion (Soutschek et al., 2017). Therefore, we hypothesized that women show stronger inequity aversion than men, with the striatum and amygdala playing a critical role.

To test these hypotheses from a neuro-cognitive point of view, we conducted a model-based fMRI study and a large-scale online behavioral study of the trust game task, which was designed to measure guilt aversion and inequity aversion. The fMRI study investigated the neural and network mechanisms for the guilt and inequity aversions, with particular focus on gender differences. For the online behavioral data, a regression analysis of guilt aversion was conducted based on Big Five and social factors, such as age and socioeconomic status, by which we expected cognitive and societal aspects of guilt aversion would be revealed.

## Materials and Methods

### Intersection of fMRI and online studies

#### Trust game

**Figure 1.**
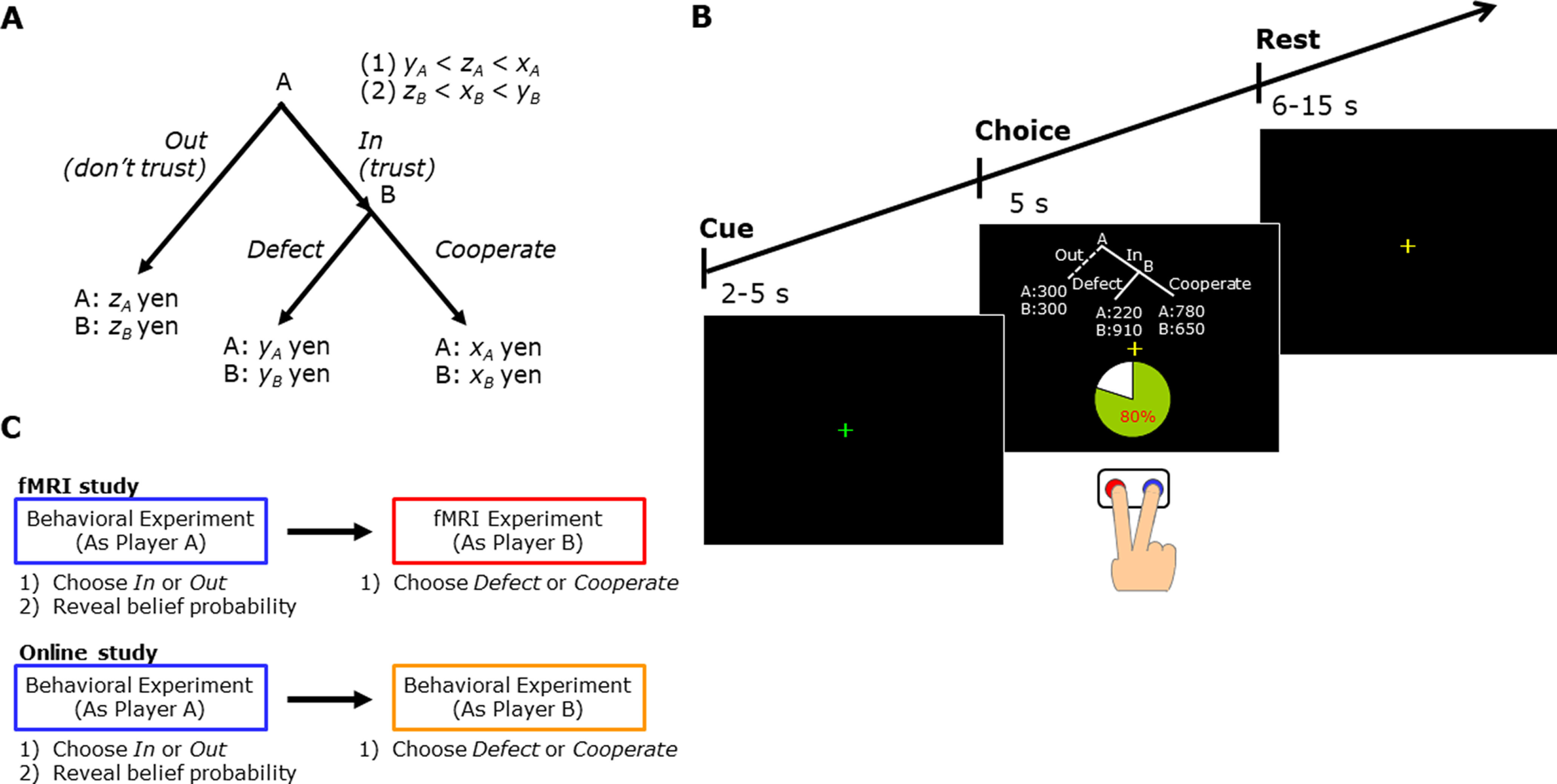
Task design. ***A***, Design of the trust game. First, player A chooses In or Out, which reveals a belief probability of the likeliness that player B will choose Cooperate. If player A chooses Out (i.e., does not trust player B), player A and B receive $z_{A}$ and $z_{B}$, respectively. If player A chooses In (i.e., trusts player B), then with the knowledge of player A’s belief probability, player B decides whether to Cooperate or Defect. If player B chooses Defect, players A and B receive $y_{A}$ and $y_{B}$, respectively; if Cooperate, players A and B receive $x_{A}$ and $x_{B}$, respectively. The actual assignment of x, y, z and $\tau_{A}$ for the 45 trials is shown in Extended Data Figure 1-1. ***B***, An outline and example of experimental trials. After the green fixation period (2–5 s; cue phase), a task condition is presented for 5 s (choice phase), and participants are asked to press the Cooperate or Defect button (blue and red, respectively). Then, a yellow fixation cross is shown for 6–15 s (rest phase). ***C***, An illustration of the complete experimental paradigm. For both the fMRI and online studies, in the first experiment, participants (as player A) chose In or Out and reveal their belief probability that player B would choose Cooperate. In the second experiment, participants (as player B) chose to Cooperate or Defect. Participants make their decisions while being scanned in the fMRI experiment. Instructions for the first and second experiments are shown in Extended Data Figure 1-2.

Participants performed a trust game adapted from the task originally used by Charness and Dufwenberg (2006). In this task, two subjects are paired as players A and B (Fig. 1*A*). First, player A must choose between In and Out options and simultaneously reveal their belief about $\tau_{A}$ (from 0% to 100%), the probability that player B will choose Cooperate. In other words, $\tau_{A}$ is player A’s level of trust in player B. If player A chooses Out, players A and B receive payments $z_{A}$ and $z_{B}$, respectively. If player A chooses In, then knowing player A’s belief probability, player B must choose Cooperate or Defect. If player B chooses Defect, player A receives $y_{A}$ and player B receives $y_{B}$; if player B chooses Cooperate, then the two players receive $x_{A}$ and $x_{B}$, respectively. In the example shown in Figure 1*B*, the belief probability of player A was 80%. If player B defected, player A and player B would receive 220 and 910 yen, respectively; if player B cooperated, they would receive 780 and 650 yen, respectively.

10.1523/ENEURO.0226-21.2021.f1-1 Extended Data Figure 1-1 The actual assignment of x, y, z and $\tau_{\text{A}}$ for the 45 trials. Download Figure 1-1, DOCX file.

**Figure 2.**
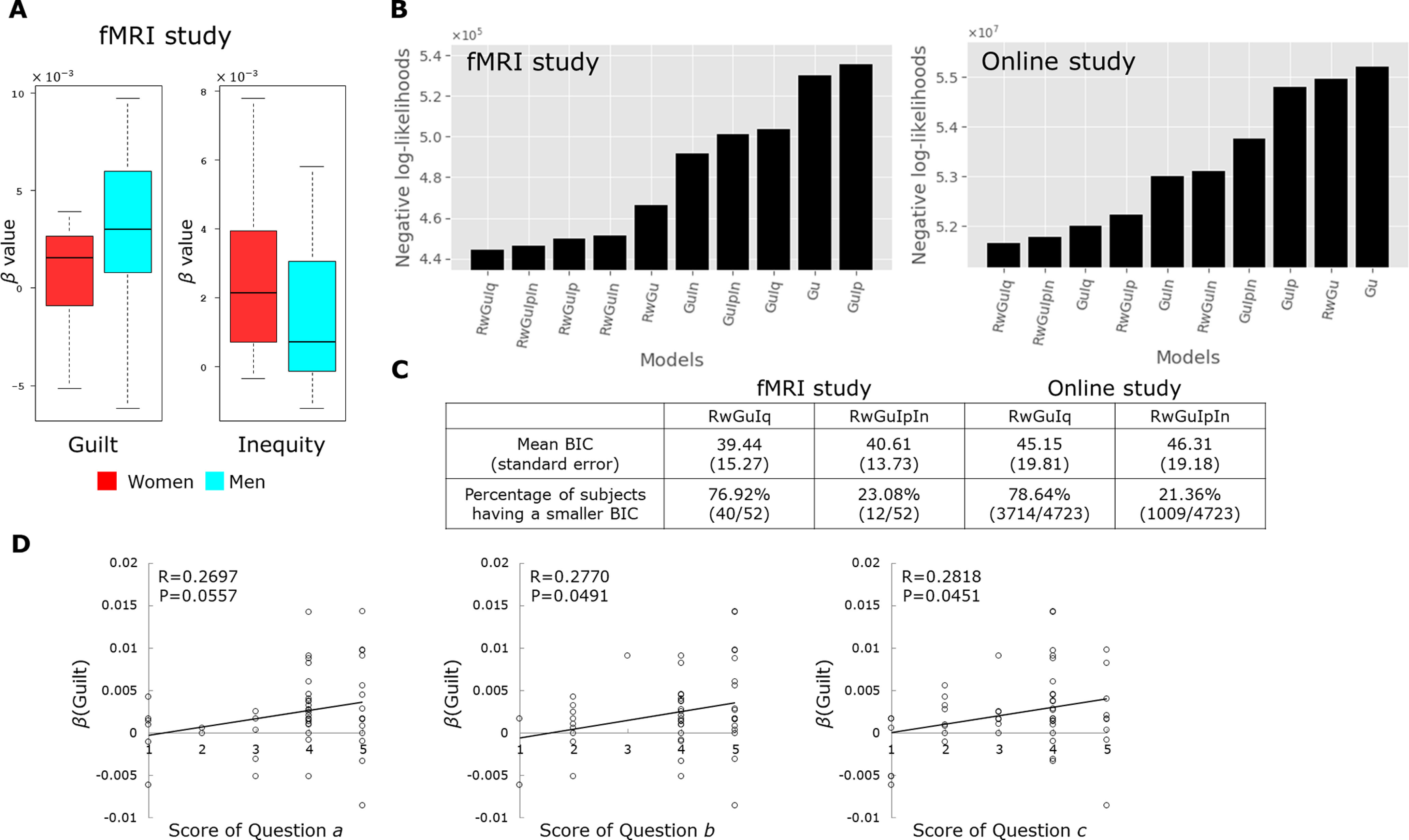
Behavioral results. ***A***, In the fMRI study (*n *=* *26 men, 26 women), the β value for guilt was higher in men than in woman (*p *=* *0.046, *t* test), whereas the β value for inequity was higher in women than in men (*p *=* *0.039, *t* test). ***B***, We validated and compared the performance of 10 models using the repeated 3-fold cross-validations and found that the model containing three predictors (Reward, Guilt, and Inequity) was best for both fMRI and online studies. Rw: Reward; Gu: Guilt; Iq: Inequity; Ip: Inequity-positive; In: Inequity-negative. ***C***, BIC also selected the same model (i.e., RwGuIq in ***B***), with the second best being the Fehr and Schmidt type model (i.e., RwGuIpIn in ***B***). For the selected model, a majority of participants exhibited the smallest BIC value for both the fMRI and online experiments. ***D***, β(Guilt) had a significantly or marginally positive correlation with questions a, b, and c.

10.1523/ENEURO.0226-21.2021.f1-2 Extended Data Figure 1-2 Instructions for the first and second experiments in the fMRI study. Download Figure 1-2, DOCX file.

There are two important conditions regarding the payments in Figure 1*A* (see also the definitions of guilt and inequity aversion below): if (1) $y_{A}<z_{A}<x_{A}$, then player A signals trust (cooperation) to player B when player A chooses In; if (2) $z_{B}<x_{B}<y_{B}$, then player B feels guilt on disappointing player A relative to player A’s belief in what player A will receive. This trust game was originally designed and used in Nihonsugi et al. (2015).

#### Guilt aversion and inequity aversion

Guilt aversion (Charness and Dufwenberg, 2006; Battigalli and Dufwenberg, 2007, 2009) assumes that an individual dislikes not meeting another’s belief. Note that guilt sensitivity elicited in the trust game by guilt aversion theory is fundamentally related to the Test of Self-Conscious Affect-3 (TOSCA-3) and the Guilt and Shame Proneness Scale (GASP), which is a common measure of guilt sensitivity in psychology, but is unrelated to shame (Bracht and Regner, 2013; Bellemare et al., 2019).

This model includes social pressure on player B if the profile (In, Defect) is played (Fig. 1*A*). Player B is assumed to believe that if player A chooses In, then player A believes that he will get a return of $\tau_{A}\cdot x_{A}+(1-\tau_{A})\cdot y_{A}$, because the setting of player A’s payoff is $y_{A}<z_{A}<x_{A}$. The difference, $\left\{\tau_{A}\cdot x_{A}+\left(1-\tau_{A}\right)\cdot y_{A}\}-y_{A}=\tau_{A}(x_{A}-y_{A}\right)$, which is non-negative in our settings, can measure how much player B believes that he/she has disappointed player A relative to player A’s belief had player B chosen Defect. In other words, the difference $\tau_{A}(x_{A}-y_{A})$ is the amount of guilt that player B experiences. Let us assume that $\gamma_{B}$ is the parameter that measures player B’s sensitivity to guilt. A player is guilt-averse and will Cooperate if $y_{B}-\gamma_{B}\cdot \tau_{A}(x_{A}-y_{A})<x_{B}$. In the example trial in Figure 1*B*, if $910-\gamma_{B}\cdot 0.8\cdot (780-220)<650$, player B will choose Cooperate. Since γ_B_ does not directly measure guilt experiences or emotional traits, we can only infer that “γ_B_ expresses sensitivity of guilt.” As mentioned in Results, however, our interpretation that γ_B_ expresses a guilty experience is consistent with the results of the postexperiment questionnaire.

By contrast, inequity aversion assumes a social preference for equitable payoffs (Fehr and Schmidt, 1999). An individual is inequity-averse if, in addition to their monetary self-interest, their utility decreases when the allocation of monetary payoffs is different. If an inequity-averse player suffers from inequity, they will choose an option that results in a smaller difference between their own and the other’s monetary payoffs. Notably, the advantageous-inequity (receiving a larger reward than others) in Fehr and Schmidt’s inequity-aversion model is also referred to as “guilt.” However, it is important to note that this outcome-based “guilt” and the intension-based “guilt” we treat in guilt-aversion are completely different.

As mentioned below, based on the results of the model selection using both the cross-validation analysis (predictive likelihood) and the Bayesian information criterion (BIC; Fig. 2*B*,*C*; see also below, Model validation and comparison), the absolute difference for inequity was found superior than the standard inequity aversion model, which splits the inequity into positive and negative terms, in the present study.

We integrated guilt aversion and inequity aversion into a utility function ( $u_{B}$) for player B as follows:

$$u_{B}\;=\{\begin{array}{c} x_{B}-\alpha_{B}\left|x_{A}-x_{B}\right|if\;the\;profile\left(In,Cooperate\right)\\ y_{B}-\gamma_{B}\cdot \tau_{A}\cdot \left(x_{A}-y_{A}\right)-\alpha_{B}\left|y_{A}-y_{B}\right|if\;the\;profile\left(\begin{array}{c} In,Defect \end{array}\right), \end{array}$$

where $\alpha_{B}$ is a constant that measures player B’s sensitivity to inequity. A narrowly self-interested agent is given the special case $\gamma_{B}=\alpha_{B}=0$. In our game, players choose between binary actions that yield two different monetary payoff allocations, $X=\left(x_{A},\;x_{B}\right)$ and $Y=\left(y_{A},\;y_{B}\right)$. The utilities of these allocations are given by the formula above, yielding $u_{B}(X)$ and $u_{B}(Y)$.

#### Statistical analysis of behavioral data

We estimated three separate components, monetary self-interest, guilt, and inequity, for each participant based on the logistic model of stochastic choice. The probability that player B chooses Cooperate can be expressed as $P_{B,Cooperate}=1/1+e^{-\{u_{B}\left(X\right)-u_{B}\left(Y\right)\}}$. Although our model does not include an inverse temperature parameter explicitly, this does not imply the model does not consider decision noise. In fact, our model implicitly assumed the inverse temperature parameter to be 1. Such an implementation of the softmax function with the inverse temperature parameter = 1 is often seen in the behavioral analysis of the economic decision-making (Boorman et al., 2009; Cai, and Padoa-Schioppa, 2014; Suzuki et al., 2015) because the inverse temperature is relatively difficult to estimate. Based on this logistic model, we used a logistic regression as follows:

$$logit(P_{B,Cooperate})=\beta_{0}+\beta_{1}Reward_{t}+\beta_{2}Guilt_{t}+\beta_{3}Inequity_{t},$$

where $Reward_{t}$ is the size of the reward and calculated as $x_{B}-y_{B}$ at time *t*, $Guilt_{t}$ is the size of guilt and calculated as $-\{0-\tau_{A}\cdot (x_{A}-y_{A})$}, and $Inequity_{t}$ is the size of inequity and calculated as $-(\left|x_{A}-x_{B}\right|-\left|y_{A}-y_{B}\right|)$. For convenience, $\beta_{1}$, $\beta_{2}$, and $\beta_{3}$ are denoted as β(Reward), β(Guilt), and β(Inequity), respectively. In order to orthogonalize the three explanatory variables, the actual $\tau_{A}$ used in the experiments was also set by the experimenter. Player B was asked to make decisions assuming that player A chose the In option. We therefore set $\tau_{A}$ to 60% or higher (player A is expected to choose the Out option when *τ* is small). More specifically, $\tau_{A}$ was 60% 7 times, 70% 5 times, 80% 13 times, 90% 11 times, and 100% 9 times. We display the actual values of x, y, z, and $\tau_{A}$ in Extended Data Figure 1-1. The correlation coefficients among the three explanatory variables were less than 0.30 and insignificant (*p *>* *0.05); the values of guilt and inequity were designed to be orthogonal [the correlation coefficient of these two variables was −0.138 and nonsignificant (*p *=* *0.367)] to dissociate the computational processes for guilt aversion and inequity aversion.

This logistic regression was computed using the R statistical package (R Core Team, 2021). We used the brglm package to conduct our maximum likelihood estimation with the bias-reduction method (Kosmidis, 2019).

#### Model validation and comparison

Our utility model comprises three separate components: Reward, Guilt, and Inequity, as defined above. With regard to Inequity, we adopted the absolute difference for Inequity. However, participants may alternatively use Fehr and Schmidt’s model, which splits the inequity into positive (called Inequity-positive hereafter) and negative (called Inequity-negative hereafter) terms. Therefore, we need to verify which model (component) better explains the data for the current experiments.

To address this issue, we first compared 10 possible models (for details of the 10 models, see Fig. 2*B*) based on the predictive negative log likelihoods using a cross-validation. This cross-validation approach for value-based decision-making allows us to avoid overfitting the data and to compare models with different numbers of parameters robustly. It has also been adopted in many recent studies (Daw, 2011; Smith et al., 2014; Linderman and Gershman, 2017; Park et al., 2019; Fig. 2*B*). We also compared more familiar BIC values for the models and exemplified the ones with the first and second minimum BIC to confirm the results (Fig. 2*C*).

More specifically, to compute the minimum predictive negative log-likelihood, we repeated bootstrap (500 iterations) 3-fold cross-validations for the model validation and comparison. For each model, we randomly divided 45 trials for each participant into three groups of equal size (i.e., 15). We fitted the model to 30 trials and predicted the behavior in the held-out 15 trials and repeated this process three times. We repeated this 3-fold cross-validation procedure 500 times and selected the model with the minimum predictive negative log-likelihood for held-out trials.

### fMRI study

#### Participants

A total of 52 participants (mean age 21.2 years; SD = 1.4 years; 26 females) participated in the fMRI experiments. They were scanned on a Siemens 3T Trio scanner at the Center for Information and Neural Networks (CiNet) of the National Institute of Information and Communications Technology (NICT). The ethical committees of the NICT approved this study, and all participants gave informed consent. Participants received money proportional to the number of payoffs earned during the experiment (equivalent to 45–60 United States dollars). Although our task was the same as the one in Nihonsugi et al. (2015), we collected completely different participants in this study for two main reasons. First, we had access to a 64-channel MRI coil to analyze the DMPFC and VMPFC. The 64-channel brain coil provides a 1.3-fold higher signal-to-noise ratio in the brain cortex than the 32-channel array (Keil et al., 2013). Second, the number of participants (*n *=* *42) in Nihonsugi et al. (2015) was not enough for re-analysis; Yarkoni (2009) suggested that a sample size of >50 is necessary for identifying a moderate correlation at relatively conservative thresholds. Additionally, we also wished to test whether we could replicate our previous results.

#### Experimental design and procedure

We conducted two experiments in which participants played a trust game in different roles (Fig. 1*C*; see also the instructions in Extended Data Fig. 1-2). In the first (behavioral) experiment, >10 participants per experiment were invited into a room and read instructions of the rules and procedure of the trust game. Every participant played the trust game as player A (i.e., choose In or Out and reveal belief probability $\tau_{A}$) and experienced one trial. The participants were informed that these choices would be used when player B made their choice in the second (fMRI) experiment. However, player A was not informed of player B’s identity. Participants were told that earnings for player A will be determined according to the actual outcome made by both players’ choices if A’s choice is used in the second experiment.

The second experiment was conducted on average 6 d (range = 1–10 d) after the first experiment. All participants played the game as player B (i.e., choose Cooperate or Defect with knowledge of player A’s belief probability) for 45 trials. Participants were instructed to assume that player A chose In in this experiment (the Out option is illustrated as a dashed line in Fig. 1*B*). The sequence of the trials was randomized across subjects. Participants were told that the other participant (player A) differed for each trial and that the pairings were anonymous. We did not provide any feedback to the participants during the experiment. Participants were also informed that earnings for player B will be the sum of the show-up fee and the actual outcome obtained from both players’ choices in the 45 trials.

Because there was the risk that player B felt that the other player was hypothetical rather than real, we invited >10 participants at a time into a room in the first experiment to make them realize the other’s presence and impress on them that they would have a real partner in the second experiment. In addition, when giving instructions for the second experiment, we repeatedly explained that we had conducted similar first experiments many times and that there were many player As and the partner in the second experiment was one of them. In other words, on the day of the second experiment, the participants were likely to think about other participants in the first experiment. Thus, although the experiment was hypothetical, we assume that the participants were engaged in the tasks as if they were in a real interaction. Indeed, no participant reported or even referred to the absence of their partner in a postexperiment interview.

After reading the instructions for the task and procedure, the participants were briefed about the rules of the game by the experimenter and tested to confirm that they understood the rules. They were then individually invited into the scanning room and practiced the game using the response buttons in the scanner.

Functional images were acquired as participants played the game. The timeline of a trial is shown in Figure 1*B*. Each trial began with a 2- to 5-s preparation interval during which time a green fixation cross was presented for the first 1 s and then a yellow fixation cross (cue phase) was presented for the remainder of the time. The participants were then presented with the trust game, including the allocation of monetary payoffs for each choice and player A’s belief, and selected Cooperate or Defect by pressing the corresponding button within 5 s (choice phase). In each trial, participants made their choice on the assumption that player A chose In. This was followed by the presentation of a fixation cross for a variable time period of 6–15 s (rest phase).

After scanning, all participants answered the questionnaire. For guilt aversion behavior, participants were asked to answer the following three questions on a five-point scale (1: I don’t think so,…, 5: I think so):

- Did you think that the reason why player A chose In was because they expected (and aimed) to gain $x_{A}$ yen (i.e., the result of player B choosing Cooperate)?
- Did you think that choosing Defect would reduce the payoff ( $x_{A}$ yen) expected by player A?
- Did you feel guilt that your choice of Defect would reduce the payoff ( $x_{A}$ yen) expected by player A?

Question *a* examined whether the respondent understood the partner’s intention of choosing In (the meaning behind the expectation); question *b* examined whether the respondent was aware that their choice of Defect reduces their partner’s expected payoff; and question *c* asked whether the respondent felt guilty when he/she reduced their partner’s expected payoff.

#### fMRI image acquisition

Scanning was performed on a Siemens 3T Trio scanner with a 64-channel coil at CiNet using an echoplanar imaging (EPI) sequence with the following parameters: repetition time (TR) = 3000 ms, echo time (TE) = 25 ms, flip angle = 90°, matrix = 64 × 64, field of view (FOV) = 192 mm, slice thickness = 3 mm, gap = 0 mm, and ascending interleaved slice acquisition of 51 axial slices. High-resolution T1-weighted anatomic scans were acquired using an MPRAGE pulse sequence (TR = 2000 ms, TE = 1.98 ms, FOV = 256 mm, image matrix 256 × 256, slice thickness = 1 mm). We discarded the first two EPI images before data processing to compensate for T1 saturation effects.

#### fMRI data preprocessing

SPM12 (http://www.fil.ion.ucl.ac.uk/spm) was used for the MRI data preprocessing and analysis. Preprocessing included motion correction, coregistration to the participant’s anatomic image, and spatial normalization to the standard Montreal Neurologic Institute (MNI) T2 template with a resampled voxel size of 2 mm. Coregistered EPI data were normalized using an anatomic normalization parameter. Spatial smoothing was performed using an 8-mm Gaussian kernel.

#### General analysis methods

To explore the neural basis of guilt, inequity and value difference, we performed a general linear model (GLM) analysis of the functional data. We constructed two GLM models.

##### GLM 1

To model the blood oxygen level-dependent (BOLD) signal driven by Guilt and Inequity, the two variables were convolved with a hemodynamic response function (HRF; spm_hrf function with TR equal to 3.0 s). For first level GLM analysis, the onset and duration were the onset timing of “Choice phase” and 0 s, respectively. In addition to a response-period constant regressor, we introduced (1) an HRF for Guilt and (2) an HRF for Inequity. Additional regressors modeling head motion, as derived from the realignment procedure, were included in the model. Serial autocorrelation was modeled as a first-order regressor, and data were high-pass filtered at a cutoff of 128 s.

We calculated second-level group contrasts using one-sample *t* tests to reveal the main effect of each parametric regressor within participants using the individual contrast images. To correct for multiple comparisons, we used for Guilt contrast the familywise error (FWE) correction across the whole brain at *p *<* *0.05 based on Gaussian random field theory as implemented in SPM12 [minimum cluster extent (*k*)* *>* *20 voxels, see also Extended Data Fig. 3-1 for the actual cluster size]. Since the analysis of Inequity targets small regions, such as the striatum and amygdala, we set the minimum cluster extent to 20 voxels to keep the extent size the same throughout the analysis of Guilt and Inequity. When analyzing Inequity, we used for the whole-brain analysis a threshold of *p *<* *0.001 uncorrected.

##### GLM 1.1

After calculating GLM1, a two-sample *t* test was used to compare Guilt contrast between men and women. For the whole-brain analysis, a threshold of *p *<* *0.001 uncorrected with an extent threshold of *k *=* *20 was adopted.

##### GLM 1.2

After calculating GLM1, a two-sample *t* test was used to compare Inequity contrast between men and women. For the whole-brain analysis, a threshold of *p *<* *0.001 uncorrected with an extent threshold of *k *=* *20 was adopted.

##### GLM 2

We modeled brain activity related to utility. For the first-level analysis, we entered the value difference between choice options (larger utility-smaller utility) as a parametric modulator of a regressor. The onset and duration were the onset timing of the Choice phase and 0 s, respectively. Additional regressors modeling head motion, as derived from the realignment procedure, were included in the model. Serial autocorrelation was modeled as a first-order regressor, and data were high-pass filtered at a cutoff of 128 s.

We calculated second-level group contrasts using one-sample *t* tests to reveal the main effect of each parametric regressor within participants using the individual contrast images. Additional regressor modeling of a gender-indicating variable was included in the model. We used for the whole-brain analysis a threshold of *p *<* *0.001 uncorrected.

#### Region of interest (ROI) analysis

For the Guilt contrast in GLM1, because of the lack of adequate previous neuroimaging studies and consistent imaging results for guilt aversion, we had no specific priori hypothesis and performed no ROI analysis. However, for GLM1.1 (gender difference in guilt), we did have a priori hypothesis from previous lesion studies that showed the VMPFC is involved in gender differences in social cognition (Tranel et al., 2005; Sutterer et al., 2015). Therefore, we performed a ROI analysis on whether this region survived a small volume correction at *p *<* *0.05 with an FWE correction. For Inequity contrast in GLM1 and GLM1.2, because we had a priori hypothesis from previous research that found the amygdala and striatum are involved in inequity (Haruno and Frith, 2010; Tricomi et al., 2010; Gospic et al., 2011; Crockett et al., 2013; Haruno et al., 2014; Tanaka et al., 2017) and there exists a gender difference in inequity (Soutschek et al., 2017), we employed a ROI analysis with a small volume correction (*p *<* *0.05; small volume FWE corrected). With regard to value difference in GLM2, we again had a priori hypothesis from previous research that found the VMPFC is involved in value difference (Hunt et al., 2012; Nicolle et al., 2012). Therefore, we employed a ROI analysis with a small volume correction (*p *<* *0.05; small volume FWE corrected).

The small volume of the VMPFC and DMPFC was based on a 15-mm sphere around the coordinates (*x *=* *2, *y *=* *41, *z* = −6) and (*x* = −3, *y *=* *48, *z *=* *30), because these coordinates were used in a neuroimaging study (Baumgartner et al., 2011) of social preferences similar to ours. In that study, the VMPFC coordinates were determined by averaging the peak coordinates across five neuroimaging studies (value and economic decision-making), and the DMPFC coordinates were based on a meta-analysis study on social cognition (van Overwalle, 2009). Furthermore, the VMPFC coordinates (subjective value: *x *=* *2, *y *=* *46, *z* = −8; decision stage: *x *=* *2, *y *=* *40, *z* = −8) in a previous meta-analysis (Bartra et al., 2013) are very close to the coordinates we used. The small volumes for the amygdala and striatum were defined using the WFU PickAtlas toolbox (Maldjian et al., 2003).

#### Psycho-physiological interaction (PPI) analysis

We performed two PPI analyses using the function of SPM12.

##### PPI1

**Figure 3.**
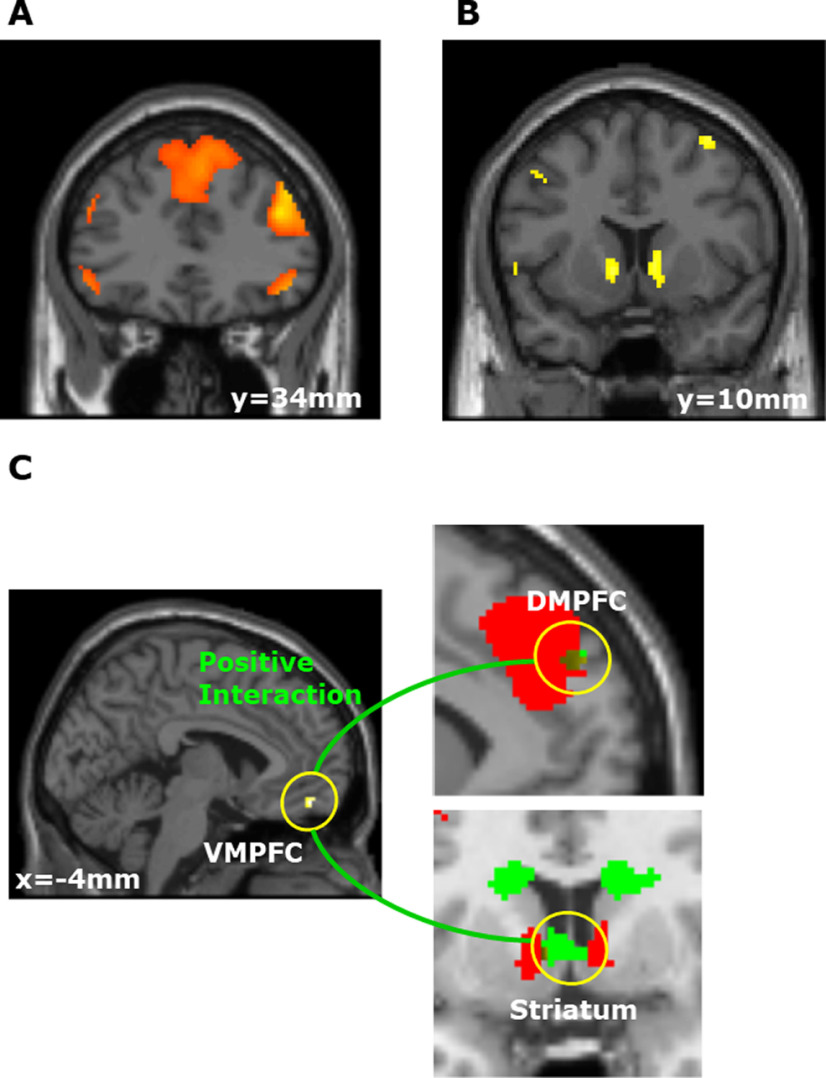
Activities correlated with Guilt, Inequity, and Utility in both genders. ***A***, Activities in the right and left DLPFC and DMPFC were correlated with guilt (right DLPFC, *p* < 0.001; left DLPFC, *p* < 0.001; DMPFC, *p* < 0.001). Activities related with Guilt in both genders are listed in Extended Data Figure 3-1. ***B***, The bilateral ventral striatum activity was correlated with inequity (right ventral striatum, *p* = 0.035; left ventral striatum, *p* = 0.042). Activities related with Inequity in both genders are listed in Extended Data Figure 3-2. ***C***, left, Activity in the VMPFC was positively correlated with the value difference (larger utility-smaller utility; *p* = 0.040, see also Extended Data Fig. 3-3). Top right, Overlay of the VMPFC × Guilt cluster (green) and the Guilt-correlated region shown in ***A*** (red). These two areas overlap in the DMPFC (brown). For display purposes, we used a threshold of *p* < 0.001 uncorrected for the Guilt contrast, and a threshold of *p* < 0.005 uncorrected for VMPFC × Guilt. Results of the PPI analysis for VMPFC × Guilt in both genders are summarized in Extended Data Figure 3-4. Bottom right, Overlay of the VMPFC × Inequity cluster (green) and the Inequity-correlated region shown in ***B*** (red). These two areas overlap in the striatum (brown) at the relaxed threshold. For display purposes, the threshold of the VMPFC × Inequity contrast is uncorrected *p* < 0.05. Results of the PPI analysis for VMPFC × Inequity in both genders are summarized in Extended Data Figure 3-5.

Having confirmed that the VMPFC was involved in value difference by the GLM2 analysis, we next conducted a hypothesis-based PPI analysis to examine whether this VMPFC activity truly integrates the value components of Guilt and Inequity. More specifically, we used VMPFC (shown in Fig. 3*C*) as a seed region and examined whether brain areas associated with VMPFC × Guilt overlapped with the Guilt-correlated areas (i.e., DLPFC and DMPFC in Fig. 3*A*) and whether brain areas associated with VMPFC × Inequity overlapped with the Inequity-correlated area (i.e., striatum in Fig. 3*B*). For each subject, we extracted the time course of activity from a 5-mm-radius volume of interest (VOI) around the peak voxel in the VMPFC (shown in Fig. 3*C*). Based on the procedure by Gitelman et al. (2003), the time series of the VOI was extracted and then deconvolved, multiplied with the psychological variable (size of Guilt or Inequity), and reconvolved with the HRF set up as the PPI regressor. The three regressors (i.e., PPI regressor, VOI time series, and psychological variable) were then convolved with the canonical HRF and entered into the regression model along with six head motion parameters. The individual parameter estimate image for the PPI regressor was subsequently subjected to a one-sample *t* test. Finally, we also included a gender-indicating variable and performed a group analysis to identify brain regions showing increased functional connectivity with the seed VOI during the Choice phase. For the whole-brain analysis, we used a threshold of *p *<* *0.001 uncorrected.

10.1523/ENEURO.0226-21.2021.f3-1 Extended Data Figure 3-1 Activities related with Guilt in both genders. Download Figure 3-1, DOCX file.

10.1523/ENEURO.0226-21.2021.f3-2 Extended Data Figure 3-2 Activities related with Inequity in both genders. Download Figure 3-2, DOCX file.

10.1523/ENEURO.0226-21.2021.f3-3 Extended Data Figure 3-3 Activities related with value differences in both genders. Download Figure 3-3, DOCX file.

**Figure 4.**
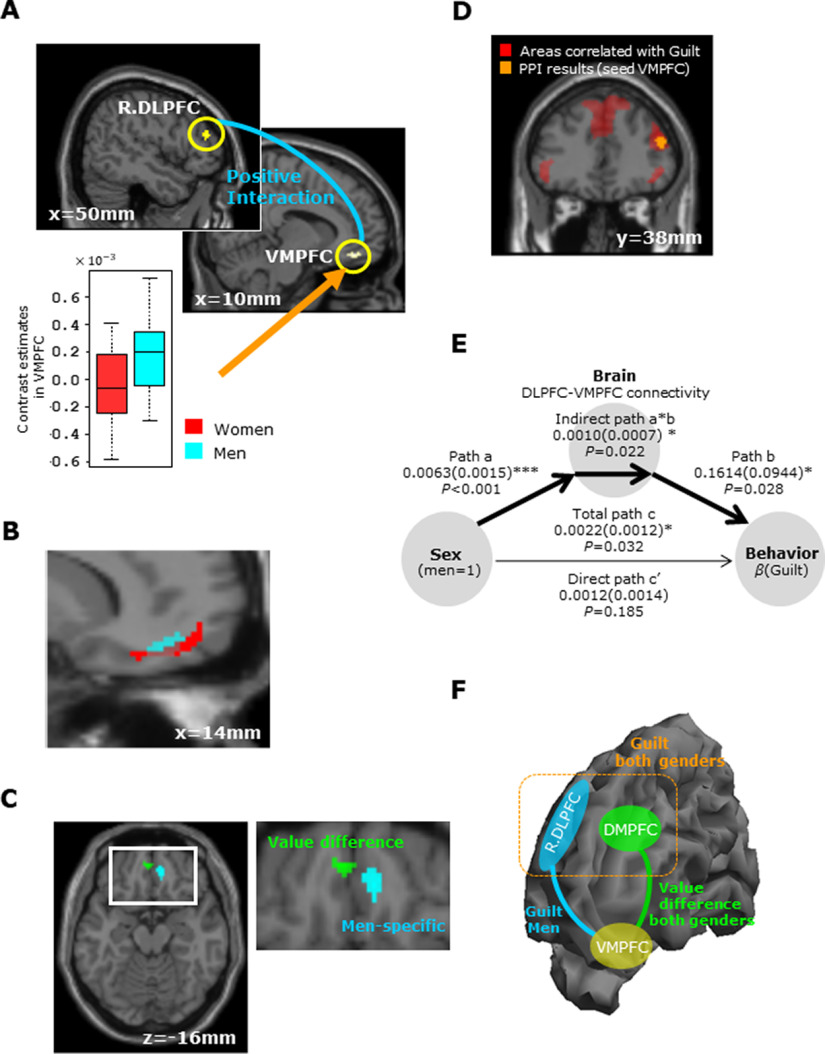
Results of gender differences for guilt in neural activity. ***A***, Men showed greater VMPFC activity than women (*p *=* *0.029). As displayed in the box plot, the extracted contrast estimates in the VMPFC demonstrate that men showed increased VMPFC activity in response to guilt (*p *<* *0.001, *t* test). Importantly, the VMPFC seed exhibited positive correlation with activity in the right DLPFC as guilt increases for men but not for women (*p *<* *0.001, uncorrected). Differences of activities related to guilt between men and women are listed in Extended Data Figure 4-1. ***B***, Overlay of the VMPFC, which is related to gender difference in Guilt (blue), and the Guilt-correlated region (red). For display purposes, the threshold for the Guilt areas is *p *<* *0.001 uncorrected and the VMPFC threshold is *p *<* *0.005 uncorrected. The activation of the VMPFC involved in gender difference in Guilt largely overlaps with the clusters of activation correlated with guilt (overlap area; brown). ***C***, Overlay of the VMPFC cluster shown in Figure 3*C*, which was positively correlated with the value difference (green), and the VMPFC cluster shown in ***A***, which showed differential activation in the guilt contrast (men > women; blue). These two areas are close but do not overlap. ***D***, Using a PPI analysis, a comparison of men and women showed enhanced functional connectivity of the VMPFC with the right DLPFC during the processing of guilt only in men (orange areas). This activation area (DLPFC) largely overlaps with the clusters of activation correlated with guilt shown in Figure 3*A* (shown in this figure as red areas). Results of the PPI analysis for guilt when testing for gender differences are shown in Extended Data Figure 4-2. ***E***, Mediation analysis of the relationship of gender, DLPFC-VMPFC connectivity and β(Guilt) shows that DLPFC-VMPFC connectivity is a complete mediator of the interaction between gender and guilt-aversion behavior. Path coefficients are shown next to arrows with SEs in parentheses; *p <* *0.05, ***p <* *0.001. ***F***, Diagram summarizing the results of our analyses. Activities in the DLPFC and DMPFC were correlated with guilt in both genders. The blue line represents a stronger connectivity between the VMPFC and right DLPFC in men than in women depending on VMPFC × Guilt, and the green line represents stronger positive coupling between the VMPFC and DMPFC depending on VMPFC × value difference.

10.1523/ENEURO.0226-21.2021.f3-4 Extended Data Figure 3-4 Results of the PPI analysis for VMPFC × Guilt in both genders. Download Figure 3-4, DOCX file.

**Figure 5.**
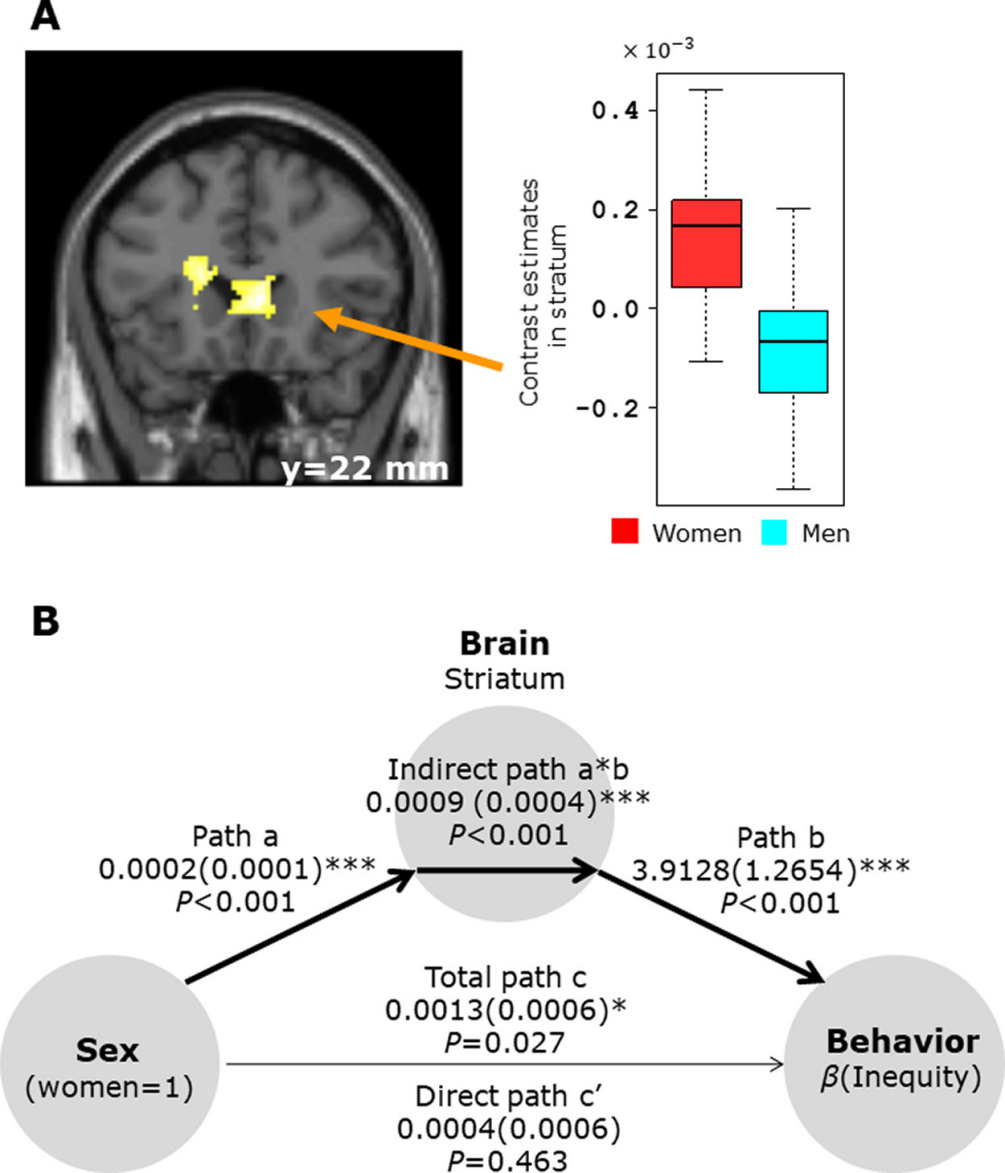
Results of gender differences in neural activity for inequity. ***A***, Women showed greater ventral striatum activity than men (*p *=* *0.008). The box plot illustrates the contrast estimates in the right ventral striatum and shows that only women showed increased activity in response to inequity (*p *<* *0.001, *t* test). Differences of activities related to inequity between men and women are summarized in Extended Data Figure 5-1. ***B***, A mediation analysis shows that the mediation effect of the striatum is significant (a*b, *p *<* *0.001). Path coefficients are shown next to the arrows with SEs in parentheses; *p <* *0.05, ***p <* *0.001.

10.1523/ENEURO.0226-21.2021.f3-5 Extended Data Figure 3-5 Results of the PPI analysis for VMPFC × Inequity in both genders. Download Figure 3-5, DOCX file.

##### PPI2

The goal of this analysis was to examine whether different brain networks are involved in the computation of guilt and inequity between men and women. More specifically, this analysis aimed to find differences between men and women in brain regions that correlate more strongly with VMPFC or striatum activity as guilt or inequity increases. For each subject, we extracted the time course of activity from VOIs with a 5-mm-radius around the peak voxel in the VMPFC, as shown in Figure 4*A*, and the ventral striatum, as shown in Figure 5*A*. For this analysis, the PPI terms were defined as VMPFC × guilt and ventral striatum × inequity. We entered six variables (i.e., PPI regressor, VOI time series and psychological variable for guilt and inequity, respectively) and movement regressors into a GLM. The individual parameter estimate image for the PPI regressor was subsequently subjected to a one-sample *t* test. Finally, group analysis was performed to identify brain regions showing increased functional connectivity with the seed VOIs. A two-sample *t* test was performed to further assess different connectivity patterns between men and women. For the whole-brain analysis, a threshold of *p *<* *0.001 uncorrected at the peak voxel level with an extent threshold of *k *=* *20 was adopted.

10.1523/ENEURO.0226-21.2021.f4-1 Extended Data Figure 4-1 Differences of activities related to guilt between men and women. Download Figure 4-1, DOCX file.

10.1523/ENEURO.0226-21.2021.f4-2 Extended Data Figure 4-2 Results of the PPI analysis for guilt when testing for gender differences. Download Figure 4-2, DOCX file.

10.1523/ENEURO.0226-21.2021.f5-1 Extended Data Figure 5-1 Differences of activities related to inequity between men and women. Download Figure 5-1, DOCX file.

#### Mediation analysis

We performed a mediation analysis to test whether the interaction between gender and guilt-based prosocial behavior was mediated by a brain function using a mediation toolbox (https://github.com/canlab/MediationToolbox; Wager et al., 2008). Briefly, this analysis was based on a standard three-variable path model, as shown in Figure 4*D*. This analysis quantifies the degree to which a relationship between two variables, X and Y, can be explained by another variable, M.

For the guilt-aversion behavioral analysis, we defined X as the gender-indicating variable (1 = men), Y as the behavioral variable, β(Guilt), and M as the brain variable functional connectivity between the right DLPFC and VMPFC (Fig. 4*A*). Following convention, we required that three tests reach statistical significance in the mediation analysis. First, path a measured the association between the gender-indicating variable and the functional connectivity. Second, path b measured the association between the functional connectivity and β(Guilt) after controlling for the gender-indicating variable. Third, the mediation effect, defined as the product of the indirect paths ( a×b), must be significant. We refer to the overall predictor-outcome relationship as effect c and the direct effect controlling for the mediator c′. Thus, the a×b effect tests the significance of c−c′. We conducted bootstrap tests (10,000 iterations) for statistical significance of the mediators.

For inequity-aversion behavioral analysis, we defined X as the gender-indicating variable (1 = women), Y as the behavioral variable β(Inequity), and M as the brain variables (striatum shown in Fig. 5*A*).

### Online study

#### Participants

We analyzed data from 4723 participants (mean age 37.9 years, SD = 15.4 years, 2737 females; for more detailed descriptive statistics, see Table 1) who followed the task instructions correctly and spent longer than 1 h to complete seven different personality trait tests such as Big Five Inventory, anxiety (STAI) and depression (BDI) and the trust game task. These data were collected using our in-house online experiment system. The study protocol was approved by the ethical committees of the NICT, and all participants gave informed consent. For their participation, participants were paid in cashable points proportional to the number of payoffs earned during the experiment (equivalent to 3–5 United States dollars).

**Table 1:** Descriptive statistics for online sample (between genders)

	Men	Women
	(*n *=* *1986)	(*n *=* *2737)
Variable	Mean (SD)	Mean (SD)
Age	39.585 (15.318)	36.751 (15.273)
Neuroticism	47.433 (9.7900)	46.731 (9.9606)
Extraversion	45.426 (9.1431)	46.244 (9.2942)
Openness	50.699 (9.4570)	47.640 (9.3566)
Agreeableness	42.780 (10.636)	44.333 (10.448)
Conscientiousness	49.068 (9.3706)	48.076 (9.3299)
SelfEduHistory	5.3197 (1.1422)	5.0431 (1.0410)
ParentsEduHistory	4.6511 (1.4190)	4.7947 (1.3323)
Income	2.7296 (1.4772)	1.6153 (0.9299)
Occupation	2.2477 (1.6859)	3.4439 (1.8253)
Subjective SES	5.2513 (2.0408)	5.2700 (1.7901)

All scores were raw values.

#### Experimental design and procedure

Participants performed a trust game on our in-house online experiment system in a similar way to the fMRI study (Fig. 1*C*). We conducted two consecutive experiments in which participants played a trust game in a different role. Before the first experiment, online participants read the rules of the trust game and the procedure. In the first experiment, every participant played the trust game as player A (i.e., choose In or Out and reveal belief probability $\tau_{A}$) and experienced one trial. Participants knew these choices would be used, and the pairings were anonymous when player B made their choice in the second behavioral experiment.

In the second experiment, all participants played the game as player B (i.e., choose Cooperate or Defect with knowledge of player A’s belief probability). Participants (player B) were instructed to assume that player A chose In. Every participant experienced 45 trials. The sequence of the trials was randomized across subjects. Participants were told that the other participant (player A) differed for each trial and that the pairings were anonymous. We did not provide any feedback to the participants during the experiment. All participants answered seven different personality trait tests including the Big Five Inventory. The final earnings were calculated following the same pattern as the fMRI study.

#### Evaluation of cognitive mechanisms using Big Five Inventory

We first examined the relationship between guilt-aversion [ β(Guilt)] and gender. Specifically, we estimated β(Guilt) for participants by the same logistic regression as the fMRI study and compared β(Guilt)s between men and women. To investigate two different cognitive processes (i.e., agreeableness and conscientiousness) potentially underlying gender difference in guilt aversion and to control for the confounding effects of the participant’s socioeconomic status, we conducted a multiple linear regression analysis based on the following equation:

$$\beta (Guilt{)}_{i}=\beta_{1}Neuroticism_{i}+\beta_{2}Extraversion_{i}+\beta_{3}Openness_{i}+\beta_{4}Agreeableness_{i}+\beta_{5}Conscientiousness_{i}+\beta_{6}Age_{i}+\beta_{7}SelfEduHistory_{i}+\beta_{8}ParentsEduHistory_{i}+\beta_{9}Income+\beta_{10}Occupation+\beta_{11}SubjectiveSES_{i}+\beta_{12}Sex_{i}\times Neuroticism_{i}+\beta_{13}Sex_{i}\times Extraversion_{i}+\beta_{14}Sex_{i}\times Openness_{i}+\beta_{15}Sex_{i}\times Agreeableness_{i}+\beta_{16}Sex_{i}\times Conscientiousness_{i}+\beta_{17}Sex_{i}\times Age_{i}+\beta_{18}Sex_{i}\times SelfEduHistory_{i}+\beta_{19}Sex_{i}\times ParentsEduHistory_{i}+\beta_{20}Sex_{i}\times Income+\beta_{21}Sex_{i}\times Occupation+\beta_{22}Sex_{i}\times SubjectiveSES_{i}+\varepsilon_{i},$$

where $Neuroticism_{i}$, $Extraversion_{i}$, $Openness_{i}$, *Agreeableness_i_*, and $Conscientiousness_{i}$ are the individual’s Big Five score (Murakami and Murakami, 1999), $Age_{i}$ is the individual’s age, $SelfEduHistory_{i}$ and $ParentsEduHistory_{i}$ are the individual’s scores of educational history and his/her parents’ score of educational history, respectively (Okada et al., 2014), $Income_{i}$ and $Occupation_{i}$ are the individual’s income and occupation, respectively (Ganzeboom et al., 1992), and $SubjectiveSES_{i}$ is the individual’s subjective socioeconomic status (Adler et al., 2000). $Sex_{i}$ is the binary variable representing individual (1)’s sex (men = 1) and used to represent interactive effects with Big Five scores and socioeconomic status variables.The multiple linear regressions were conducted using the glm package based on the R statistical package (R Core Team, 2021).

## Results

### fMRI study

#### Behavioral results of the fMRI study

We first performed a logistic regression analysis to determine whether reward, guilt, and inequity had an effect on participant behavior (Cooperate or Defect). Behavioral data from the fMRI experiment (*n *=* *52) were analyzed using the utility function, which comprises a linearly weighted sum of reward, guilt, and (absolute) inequity (for details, see Materials and Methods). The β values of the three predictors, Reward, Guilt, and Inequity, were positive and significant (*p *<* *0.001; Table 2), indicating that they all played critical roles in the current task.

**Table 2:** Logistic regression models predicting decision to cooperate or defect

	Dependent variable: *logit(P_B,Cooperate_)*	
Explanatory variable	fMRI	Online
Reward	0.0033317***	0.0098942***
Guilt	0.0014490***	0.0029833***
Inequity	0.0011182***	0.0059259***
Constant	−0.47831**	0.0973352***
McFadden’s *R*^2^	0.09147	0.01479
Observations	2340	212535

Significance: ∗∗∗*p *<* *0.001, ∗∗*p *<* *0.01.

Having confirmed that these three factors play crucial roles in the current task, we then compared β values between men and women. This analysis showed that the β value of Guilt [called β(Guilt) hereafter] of men was significantly higher (*t*_(41.6)_ = 2.05, *p *=* *0.046; Fig. 2*A*) than that of women, whereas the β value of Inequity [called β(Inequity) hereafter] of women was significantly higher (*t*_(48.7)_ = 2.11, *p *=* *0.039; Fig. 2*A*) than that of men. These findings show that gender differences in prosocial behavior are heterogeneous depending on the underlying motives.

For the model validation and selection, 10 possible models were compared based on the predictive negative log likelihoods by a cross-validation. This cross-validation approach for value-based decision-making allows us to avoid overfitting the data and to compare models with different numbers of parameters robustly; it has also been adopted in many recent studies (Daw, 2011; Smith et al., 2014; Linderman and Gershman, 2017; Park et al., 2019; Fig. 2*B*; see also Model validation and comparison in Materials and Methods). More specifically, we introduced a bootstrap sampling (500 iterations) and compared the model predictions to the held-out data across all folds based on the negative log-likelihood of the estimated model for each participant. We then selected the model with the minimum negative log-likelihood and found that the best-fit model contained three predictors: Reward, Guilt, and Inequity. In addition, we compared the BIC and found not only that the best model was the same with the smaller mean BIC than the second best model of Fehr and Schmidt (1999; 39.44 vs 40.61), but also that for 40 of the 52 participants (76.9%), the smallest BIC model was the best individual model (Fig. 2*C*).

Finally, we examined whether the guilt aversion parameter β(Guilt) reflects the guilt experience of the participants in the current experiment. Note that β(Guilt) captures a decision strategy to avoid future guilt but does not directly measure guilt. To address this issue, we analyzed the relationship between β(Guilt) and the score of the postexperiment questionnaire (see Materials and Methods for the questionnaire). Question *a* asked whether participants understood the intentions behind player A’s action, question *b* asked whether participants understood that they reduced player A’s payoff if they chose Defect, and question *c* asked whether participants felt guilty when they reduced player A’s expected payoff. We found significant or marginal positive correlation between β(Guilt) and scores for the questions (Fig. 2*D*; question *a*, *p *=* *0.0557; question *b*, *p *=* *0.0491; question *c*, *p *=* *0.0451). These results indicate that the guilt aversion parameter reflects the guilt experience in the current study.

#### Imaging results of guilt, inequity, and utility

For the imaging, we first examined the brain regions activated commonly in both genders. Similar to the logistic regression, a GLM analysis was conducted (SPM 12) to identify brain regions whose activity was correlated with the difference in guilt and inequity between the two choice options (hereafter, we call these differences guilt and inequity, respectively, for simplicity; see GLM1 in Materials and Methods). We included guilt and inequity as additional regressors attached to the task presentation event. We found a significant correlation between the amount of guilt and activity in the bilateral DLPFC and DMPFC [right DLPFC, *p *<* *0.001; left DLPFC, *p *<* *0.001; DMPFC, *p *< 0.001; family-wise error (FWE) corrected; Fig. 3*A*; Extended Data Fig. 3-1]. By contrast, the amount of inequity was correlated with activity in the bilateral ventral striatum (right ventral striatum, *p *=* *0.035; left ventral striatum, *p *=* *0.042; small volume FWE corrected; Fig. 3*B*; Extended Data Fig. 3-2). Additionally, we confirmed that the same results were obtained even when the two parameters (Guilt and Inequity) of GLM1 were analyzed as separate GLMs.

To identify the neural substrates that integrate different types of values, such as guilt and inequity, we searched for the neural correlates of the value difference between the choice options (larger utility-smaller utility; see GLM2 in Materials and Methods). We found a significant correlation between the value difference and activity in the VMPFC (*p *=* *0.040; small volume FWE corrected; Fig. 3*C*; Extended Data Fig. 3-3), which is consistent with previous neuroimaging studies of value-based decision-making (Hunt et al., 2012; Nicolle et al., 2012).

We next performed a PPI analysis (Friston et al., 1997) to confirm the value signals in the VMPFC reflect the value components of both Guilt and Inequity. In our behavioral hypothesis, because participants make decisions depending on both the guilt and inequity components, the VMPFC should link with both the guilt-correlated area (DLPFC and DMPFC shown in Fig. 3*A*) and inequality-correlated area (striatum shown in Fig. 3*B*). To validate this hypothesis, we estimated a PPI in which signals in the VMPFC were modulated by the Guilt or Inequity values separately for each condition (see PPI1 in Materials and Methods). More specifically, we used the VMPFC (shown in Fig. 3*C*) as the seed region to determine which other brain regions correlated with VMPFC × Guilt and VMPFC × Inequity, respectively. For the PPI of VMPFC × Guilt, this analysis revealed positive coupling between the VMPFC and the DMPFC (*p *<* *0.001, uncorrected; Fig. 3*C*; Extended Data Fig. 3-4). Notably, the VMPFC × Guilt contrast overlapped the guilt-correlated region in Figure 3*A* (Fig. 3*C*). On the other hand, for VMPFC × Inequity, we found positive coupling between the VMPFC and the striatum (*p *<* *0.001, uncorrected; Fig. 3*C*; Extended Data Fig. 3-5). The VMPFC × Inequity contrast overlaps the inequity-correlated region in Figure 3*B* at the relaxed threshold (Fig. 3*C*; VMPFC × Inequity, uncorrected *p *< 0.05). These results suggest that the guilt difference and inequity difference between the two options computed in the DMPFC and striatum contribute to the value difference in the VMPFC for both men and women.

#### Imaging results of gender differences for guilt

Next, we explored the different neural substrates for guilt aversion between men and women (see GLM1.1 in Materials and Methods). Men showed higher correlation with guilt in the VMPFC (*p *=* *0.029; small volume FWE corrected; Fig. 4*A*; Extended Data Fig. 4-1) compared with women, whereas there was no significant brain activity in the opposite contrast even at moderate threshold (uncorrected *p *<* *0.005). Figure 4*A* illustrates a box plot of the contrast estimate from the VMPFC, confirming that men showed increased VMPFC activity (*t*_(49.9)_ = 3.68, *p *< 0.001) when responding to guilt. Furthermore, this activation of the VMPFC overlapped with the activity correlated with guilt (Fig. 4*B*), indicating that the VMPFC is sensitive to guilt aversion overall and more so in men than in women. Importantly, the VMPFC activity correlating with the value difference was spatially close but did not overlap with the VMPFC activity correlating with the gender difference (Fig. 4*C*). This observation suggests that the two VMPFC areas are involved in related but distinct computations.

Having revealed gender differences in brain activity for guilt, we next performed a PPI analysis to examine whether different neural links work for guilt aversion in men and women. More specifically, we used the VMPFC (shown in Fig. 4*A*) as a seed region to search which other cortical regions correlated with the VMPFC × Guilt and then conducted two-sample *t* tests to compare this contrast between men and women (see PPI2 in Materials and Methods). In other words, the aim of this analysis was to find differences between men and women in brain regions whose activity correlate more strongly with VMPFC activity in accordance with the increase of guilt. This analysis revealed that connectivity between the VMPFC and the right DLPFC is significantly stronger in men than in women (*p *<* *0.001, uncorrected; Fig. 4*A*; Extended Data Fig. 4-2). The active right DLPFC area overlapped with the common activity correlated with guilt for men and women (Fig. 4*D*), suggesting that men recruit DLPFC-VMPFC connectivity more for guilt aversion, although the DLPFC works with the DMPFC to compute guilt in both genders.

The results so far suggest the possibility that the relationship of gender and guilt aversion is mediated by DLPFC-VMPFC connectivity. We therefore performed a mediation analysis to examine this hypothesis (Fig. 4*E*; see Materials and Methods). Figure 4*E* shows the results of this analysis and suggests that DLPFC-VMPFC connectivity is a complete mediator of the interaction between gender and guilt-aversion behavior.

In summary, according to our PPI and mediation analyses, the DMPFC works with the DLPFC to compute guilt for both genders, and the VMPFC encodes not only the value difference in collaboration with the DMPFC in both genders but also the amount of guilt (difference) in collaboration with the DLPFC predominantly in men (Fig. 4*F*).

#### Imaging results of gender difference for inequity

We also searched for gender-related neural substrates for inequity aversion (see Materials and Methods, GLM1.2). We found that the ventral striatum was significantly more active in women than in men (*p *=* *0.008; small volume FWE corrected; Fig. 5*A*; Extended Data Fig. 5-1), but there was no significant brain activity in the opposite contrast even at moderate threshold (uncorrected *p *<* *0.005). The box plot of the contrast estimates in the ventral striatum (Fig. 5*A*) demonstrates that activity in this region was correlated with the increased inequity in women (*t*_(50.0)_ = 4.26, *p *<* *0.001). When we computed PPI for functional connectivity between the ventral striatum (Fig. 5*A* as the seed region) and other brain areas in correlation with ventral striatum × inequity (see Materials and Methods, PPI2), no differential link was identified between men and women (at uncorrected *p *<* *0.001), indicating the important role of the ventral striatum in inequity aversion. Indeed, we performed a mediation analysis for our hypothesis that the relationship of gender and inequity-aversion behavior is mediated by the ventral striatum (Fig. 5*B*; see also Materials and Methods, Mediation analysis) and found that the mediation effect of the striatum is significant (a*b, *p *<* *0.001).

### Online study

The behavioral data of our fMRI study (*n *=* *52) showed that men display greater guilt aversion than women. However, this analysis provided only weak evidence because it was based on a relatively small dataset. In addition, our fMRI results did not specify the cognitive processes underlying the gender differences in guilt aversion, although the DLPFC-VMPFC connectivity result suggested a possibility that social norms play a key role, as discussed below. To clarify these issues and make the results more robust, we conducted a large-scale online behavioral study that also considered Big Five Inventory scores (Costa and McCrae, 1992) and socioeconomic status.

The differential use of prefrontal networks during guilt aversion may reflect different cognitive strategies used by men and women. Guilt aversion requires the ability to assess another individual’s expectations and directly relates to his or her disappointment (i.e., empathy or theory of mind; Hoffman, 1982). On the other hand, guilt aversion is also a normative behavior elicited by experience (Haidt, 2003) and therefore may be executed by self-discipline without requiring empathy or inference about another’s mind (i.e., rule-based decisions or systemizing). Thus, we can think of two potential cognitive underpinnings of guilt-based prosocial behavior: empathy with the disappointment of others and rule-based decisions by self-discipline. Related to this, previous studies have reported that the link between the DMPFC and VMPFC and the one between the DLPFC and VMPFC are involved in the theory of mind (De Martino et al., 2013) and in social norms (Baumgartner et al., 2011; Pornpattananangkul et al., 2018; Hackel et al., 2020) and self-control (Hare et al., 2009; Steinbeis et al., 2016), respectively. However, it is also important to be careful of reverse inference.

Because evidence connecting these prefrontal networks and gender differences in guilt aversion remain elusive, we further investigated this issue using the Big Five Inventory (Costa and McCrae, 1992), which defines five fundamental dimensions of personality (i.e., neuroticism, extraversion, openness, agreeableness, and conscientiousness). Because agreeableness is characterized by the understanding of others’ emotions, intentions and mental states, and conscientiousness is characterized by rule-based regulation and self-discipline (DeYoung et al., 2010), we hypothesized that guilt aversion correlates with agreeableness and conscientiousness and may also explain gender differences.

#### Behavioral results of online study

We first conducted a model selection using the same cross-validation analysis as the fMRI study and found that as in our fMRI study the same model containing three predictors: Reward, Guilt, and Inequity (Fig. 2*B*; see also Materials and Methods) was selected as the best model. The same result was also found by the BIC analysis (Fig. 2*C*). We then performed the logistic regression comprised of reward, guilt, and inequity and found that the β values of Reward, Guilt, and Inequity were positive and significant (*p *<* *0.001; Table 2), indicating that they all played a critical role in the online experiment.

To identify the relationship between guilt aversion [ β(Guilt)] and gender, we first performed a GLM analysis based on the explanatory variables including the gender term (Sex; men = 1), Big Five and socioeconomic status scores [target variable: β(Guilt)] for all participants. The coefficients of Sex was positive and significant (*p *<* *0.001; for other significant coefficients, Agreeableness and Income, *p *<* *0.001), demonstrating that men displayed greater guilt aversion than women, validating the behavioral result in the fMRI study with even larger data.

Next, to identify the cognitive mechanisms specific to either gender, we performed the second GLM analysis that included interaction terms between the gender variable sex and Big Five and socioeconomic status scores (for more details, see Materials and Methods). We found that the coefficients of Agreeableness and Sex × Conscientiousness were positive and significant (Agreeableness, *p *=* *0.00,802; Sex × Conscientiousness, *p *=* *0.00,712; see Table 3). These findings support our hypothesis that for guilt aversion, both men and women use the empathic strategy, while men also recruit the rule-based strategy (i.e., social norms).

**Table 3:** GLM analyses of guilt

	Dependent variable: β value for Guilt	
Explanatory variable	Coefficient	SEs
Neuroticism	0.0002667	0.0006187
Extraversion	−0.0008909	0.0006630
Openness	0.0004214	0.0007000
Agreeableness	0.0016424**	0.0006193
Conscientiousness	0.0009482	0.0006558
Age	0.0006831	0.0006325
SelfEduHistory	−0.0007013*	0.0005958
ParentsEduHistory	0.0006947	0.0006141
Income	−0.0001091	0.0008564
Occupation	0.0008336	0.0007700
SubjectiveSES	−0.0014377*	0.0006257
Sex × Neuroticism	0.0019212	0.0029112
Sex × Extraversion	−0.0020649	0.0032372
Sex × Openness	0.0004377	0.0036139
Sex × Agreeableness	−0.0035545	0.0025872
Sex × Conscientiousness	0.0089586**	0.0033272
Sex × Age	−0.0028621	0.0016136
Sex × SelfEduHistory	−0.0005493	0.0026958
Sex × ParentsEduHistory	0.0008173	0.0020632
Sex × Income	−0.0013636	0.0015921
Sex × Occupation	0.0002769	0.0013028
Sex × SubjectiveSES	−0.0005761	0.0018053
Adjusted *R*^2^	−0.07467266	
Observations	4723	

Significance level: *0.05, **0.01.

## Discussion

In this study, in correspondence with stronger guilt aversion in men than women, we demonstrated that men recruit DLPFC-VMPFC connectivity more in the processing of guilt than women do. We also found that the DMPFC is involved in the processing of guilt and the value difference between the choice options for both men and women. The analysis of the online behavioral data of 4723 participants not only replicated the gender difference in guilt aversion, but also suggested that the stronger guilt aversion in men than women is attributable to the use of rule-based (social norm-based) strategies more, while both genders commonly use empathetic consideration. Previous behavioral economics studies have closely examined guilt aversion in social interactions (Charness and Dufwenberg, 2006; Khalmetski, 2016; Bellemare et al., 2017, 2018), but to our knowledge, this is the first study reporting the evidence of gender differences in guilt aversion. Additionally, we also replicated a previously reported result (Soutschek et al., 2017) that women show greater activity of the ventral striatum than men for stronger inequity aversion.

For inequity-based prosocial behaviors, previous behavioral studies have reported that men choose efficient allocations, while women are more inequality-averse (Croson and Gneezy, 2009; Kamas and Preston, 2015). In the ultimatum game, women are significantly more likely to propose an equal split than men (Güth et al., 2007) and more likely to reject lower offers than men (Solnick, 2001). Furthermore, in the dictator game and social value orientation tasks, women are more inequality-averse in their dictator-giving (Bolton and Katok, 1995; Eckel and Grossman, 1998; Andreoni and Vesterlund, 2001; Dickinson and Tiefenthaler, 2002; Grosch and Rau, 2017). With regard to brain function, previous studies have reported a key role of the ventral striatum in resource allocation and inequity aversion. Not only is ventral striatum activity positively correlated with the ratio of the payoff (i.e., the self’s payoff vs the other’s payoff; Fliessbach et al., 2007), it is also activated when inequity between the self and the other is reduced (Tricomi et al., 2010) and when making a decision to punish someone for acting unfairly (Crockett et al., 2013). A recent study suggested that activation patterns of the ventral striatum are gender-specific, being more sensitive to sharing money with others in women (Soutschek et al., 2017). The present study is consistent with these previous studies in the sense that women show stronger inequity aversion than men, with the ventral striatum playing a critical role.

At the neural level, previous studies have reported that DLPFC and DMPFC activity varies with guilt (Chang et al., 2011; Nihonsugi et al., 2015; van Baar et al., 2019). With regard to gender differences, the current study showed that the VMPFC plays a critical role in computing guilt in men. The VMPFC has been implicated in social cognition (Blakemore, 2008). For instance, the VMPFC was implicated in affective regulation and depression (Ressler and Mayberg, 2007), the evaluation of moral dilemmas (Crockett et al., 2017), and social value decision-making (Hare et al., 2010; Baumgartner et al., 2011). In line with these studies, some studies showed that men with right VMPFC lesions have deficits in social emotion and decision-making compared with men with left VMPFC lesions, but no such difference was seen in women (Tranel et al., 2005; Sutterer et al., 2015). In addition, men with right VMPFC lesions tended to show a significant elevation in paranoia and introversion according to the Minnesota Multiphasic Personality Inventory-2 Scale, a widely-used measure of personality and psychopathology (Tranel et al., 2005). These results suggest that the right VMPFC plays an important role in social decision-making in men, consistent with the present study reporting that men use the right VMPFC (coordinates 10, 42, −16) more than women to implement guilt aversion.

A previous study showed that connectivity between the VMPFC and DLPFC in men is associated with normative decisions in the ultimatum game (Baumgartner et al., 2011). The study recruited male subjects (*n *=* *32) and demonstrated that repetitive transcranial magnetic stimulation applied to the right DLPFC of responders in the ultimatum game subsequently reduced their rejection rate (i.e., normative decision) and also diminished activity in the DLPFC and VMPFC. This result is consistent with our view that connectivity between the VMPFC and DLPFC plays a key role in guilt-based prosocial behavior in men.

For the cognitive mechanisms underlying gender differences in guilt aversion, our online study showed that guilt aversion in men correlates with conscientiousness. The Empathizing-Systemizing theory is widely known as a measure of individual differences in cognition (Baron-Cohen et al., 2003). Empathizing is the drive to identify another’s mental state and to respond with an appropriate emotion and has a positive correlation with agreeableness (Nettle, 2007; Wakabayashi and Kawashima, 2015). On the other hand, systemizing is defined as the drive to analyze, understand, predict, control, and construct rule-based systems (e.g., map-reading, physics, and mathematics) and has a positive correlation with conscientiousness, which has a desire for order as one of its components (Nettle, 2007; Wakabayashi and Kawashima, 2015). Interestingly, several previous studies showed that men are more interested in systemizing than women (Baron-Cohen, 2004; Greenberg et al., 2018). These behavioral backgrounds are consistent with our functional connectivity result of the DLPFC-VMPFC, as this link has been associated with social norms (Baumgartner et al., 2011; Pornpattananangkul et al., 2018; Hackel et al., 2020). It may also be worth noting that our results suggest that guilt aversion contains both empathizing (empathy or theory of mind) and systemizing (social norms) components. By conducting a large-scale online behavioral study, we strengthened the neuroscientific hypothesis that the DLPFC-VMPFC connectivity predominantly seen in men contributes to their stronger guilt aversion by the influence of social norms. Because the recruitment of an equally large sample for fMRI experiments is very difficult, we believe that integrating fMRI and large-scale online experiments provides a powerful tool to obtain broader and more reliable insights into human cognitions.

There is the possibility that a small *τ* may elicit an emotion other than guilt, such as distrust, because we did not directly measure emotions to belief ( $\tau_{A}$). Distrustful behavior can be perceived as hostile acts and reduce cooperation (Fehr and Rockenbach, 2003). Related to this, a previous behavioral study (Balafoutas and Fornwagner, 2017) showed that there is an inverted-U shape relationship between belief and guilt aversion using a simple dictator game. This relationship suggests that there is a threshold beyond which guilt aversion no longer applies and higher perceived expectations lead to less kind behavior on the part of the decision makers. However, this phenomenon may only occur in the dictator game, because the dictator is less likely to feel guilt because of the lack of a rational reason to live up to the recipient’s expectations. In any case, the fact that some previous research findings did not show a linear relationship between belief (*τ*) and cooperation is likely to reflect psychological differences in response to the size of belief. The present study did not allow us to address these issues, because it only considered reasonably high belief ( $\tau_{A}$). Future research should assess emotions to beliefs more precisely.

Finally, our findings do not preclude the possibility that social environments largely contribute to the gender differences in guilt aversion instead of biological reasons. At the same time, our behavioral data (*n *=* *4723) suggested that gender differences in guilt aversion are independent of age (see Table 3), indicating that gender differences are only weakly dependent on contemporaneous social environmental factors and more affected by long-lasting determinants such as social systems and biological factors. Therefore, further investigation is necessary to address what causes the gender differences in guilt aversion. For instance, we need to examine the behavioral and neural gender differences in guilt aversion in different cultures (i.e., South-East Asian and European countries). Such studies would provide more biological and societal insights into our understanding in the diversity of human prosocial behaviors.

## Synthesis

Reviewing Editor: Julie Bakker, University of Liege Decisions are customarily a result of the Reviewing Editor and the peer reviewers coming together and discussing their recommendations until a consensus is reached. When revisions are invited, a fact-based synthesis statement explaining their decision and outlining what is needed to prepare a revision will be listed below.

In this manuscript, the authors reported an fMRI study and a large-scale behavioral study that examine the gender difference in guilt aversion. They found that men and women participants relied on both shared and distinct neurocognitive processes in trust-related decision-making. Neurally, they found that both DMPFC and DLPFC were associated with a guilt-aversion cognitive computation. The functional connectivity between one of the guilt-aversion related brain region (DLPFC) and a value related brain region (VMPFC) differentiated men and women. The authors also reported a large-scale behavioral study, which revealed that some personality traits may exhibit differential correlates with guilt aversion in men and women. Overall, the work reported in the manuscript has the potential to advance our understanding of the neural correlates of guilt aversion. As a disclaimer, a reviewer had previously reviewed this manuscript for another journal, but was glad to see that the authors have addressed some of the issues raised by those reviews. That said, the reviewer felt that some important methodological details still need to be spelled out more clearly.

Major points:

1. The fMRI task looks very similar (if not identical) to the task the authors reported in their 2015 paper. We assume that the participants in this study were a completely different group of participants than those reported in their 2015 paper. Please confirm that this is the case. If not, the authors should clearly state that this is a re-analysis of a published dataset. If, as assumed, this study was based on a completely separate group of participants, then a remaining question is, what considerations justified the new data collection, given that most of the analyses reported in the current manuscript (e.g., gender difference, functional connectivity) can be done by re-analyzing their existing dataset.

2. Some important details of the task are still missing.

a. It was unclear where the authors describe how the τ parameter was determined. The authors mentioned that this parameter was pre-determined by the experimenters, but no details seem to be provided. A more conceptual issue related to this parameter is that when τ is very low (e.g., 50%, 60%), participants may interpret that as distrust. Such an interpretation may completely change Player B’s mentality - instead of anticipating guilt, they may actually have a desire to revenge or retaliate Player A (Fehr, E., & Rockenbach, B., 2003, Detrimental effects of sanctions on human altruism. Nature). Without direct measure of the participants’ emotions and strategies, this issue would be difficult to resolve. Nevertheless, the authors may want to discuss this possibility and speculate on what future studies can do to better address this.

b. How was participants’ payoff determined? Was one trial randomly selected and made real at the end of the experiment? How were the participants instructed regarding Player A’s payoff? They were all assigned to the role of Player A before the main task. Were they told that their choice as Player A would influence their final payoff? Perhaps more importantly, did they believe that their choices as Player B would matter to other Player As? The way the authors described this implies that they framed Player B’s task as hypothetical (e.g., “participants made their choice on the assumption that player A chose In”). If so, this need to be made more explicitly because it is very important to know whether the participants treated their choices as real or hypothetical. The authors may want to provide their full instructions as a supplemental material or to share it on OSF.

c. The way the authors described the timing of the fMRI task was a bit confusing. “Participants ... selected Cooperate or Defect by pressing the corresponding button at 5-8 s (choice phase).” Does this mean the choice phase begins at the 5th second of a trial and end at the 8th second of the trial? Or does this mean that in some trials, participants had 5 secs to respond, but in other trials they had up to 8 secs to respond? If the latter, then what was the purpose of including varying response intervals?

3. How did the authors’ models deal with decision noise? Typically, for binary choice, one would include an inverse temperature parameter in the softmax function to capture decision noise around participants’ indifference point. The way the authors described their model made implies that there was no such parameter in their model.

4. In this manuscript, the authors used the concept of ’guilt aversion’ as if it were emotion experiences or emotion trait (e.g., “γB is the parameter that measures player B’s sensitivity to guilt). While we agree that guilt aversion parameter captures a decision strategy as if Player A were avoiding future guilt. However, without direct measures of participants emotion and motivation, this is only an inference and should be stated explicitly.

5. “...we used for Guilt contrast the familywise error (FWE) correction across the whole brain at P < 0.05 based on Gaussian random field theory (k > 20 voxels)” (line 251). The authors should provide more details about this Gaussian random field method. Based on experience with SPM12, the cluster size required for an effect to pass the rather stringent whole-brain FEW correction (p < 0.05) is typically much larger than 20 voxels (assuming 3 * 3 * 3 mm voxel size).

6. In some of the GLMs, the authors include two parametric modulators (i.e., inequity aversion and guilt aversion) for a single event regressor (e.g., line 250). Are these two parametric modulators allowed to compete for variance? Would they get the same results if they modeled the parametric modulators in separate GLMs?

7. On page 12, the authors stated that the goal of PPI_1 was to “examine whether and which brain networks were involved in the computation of utility”. The only region identified by this analysis was the DMPFC, which was associated with guilt aversion. Does this mean the subjective value was only constituted by guilt aversion? Does inequity aversion not contribute to the computation of subjective utility? This seems contradict their behavioral and model-based findings. We suggest that the authors 1) run a hypothesis-driven, ROI-based PPI by examining the PPI effects in the regions associated with guilt aversion, inequity aversion, and reward (cf. Lim, S. L., O’Doherty, J. P., & Rangel, A. (2013). Stimulus value signals in ventromedial PFC reflect the integration of attribute value signals computed in fusiform gyrus and posterior superior temporal gyrus. Journal of Neuroscience), or 2) carry out a Dynamic Causal Modeling analysis, examining the information flow between the regions encoding components of value specified in their computational model (guilt aversion, inequity aversion, and reward) on the one hand, and the region encoding subjective value (i.e., VMPFC) (cf. Yu, H., Gao, X., Zhou, Y., & Zhou, X. (2018). Decomposing gratitude: representation and integration of cognitive antecedents of gratitude in the brain. Journal of Neuroscience).

8. The gender difference in guilt aversion revealed an area in VMPFC. However, we do not know whether this area is within the brain network sensitive to guilt aversion across the entire participant sample. In other words, although this area may exhibit significant difference in the response to guilt aversion between male and female participants, it is possible that this region is not sensitive to guilt aversion in either male or female participants (e.g., slightly negatively correlated with guilt aversion in female participants, and slightly positively correlated with guilt aversion in male participants). A more appropriate analysis would be to restrict the gender difference analysis within the areas identified in Fig. 3a. The areas identified by this analysis will then be sensitive to guilt aversion overall, and more so in male than in female participants.

9. Did the authors include any post-experiment manipulation checks and debriefing questionnaires (e.g., self-reported guilt, guilt proneness)? It would be theoretically important and interesting to know if the “guilt aversion” parameter actually reflects participants guilt experience or guilt proneness. Alternatively, the authors could adopt a “neural signature” approach to examine the guilt-related neurocognitive processes. For example, they could apply the guilt-related brain signature developed in a recent study their brain map associated with the guilt aversion parameter (Yu et al., 2020, A generalizable multivariate brain pattern for interpersonal guilt. Cerebral Cortex; Caspar et al., 2020, Obeying orders reduces vicarious brain activation towards victims’ pain. NeuroImage). One prediction would be, the pattern expression values of the guilt aversion map should be positively correlated with the γB parameter across participants. If this is indeed the case, then it would be a very strong evidence that guilt aversion strategy does recruit guilt-related neurocognitive processes.

10. The behavioral study seems very disconnected with the fMRI study. It does not seem clear what neuroscience implications and insights it could offer, and therefore, not align well with the goals of this journal. We suggest dropping this study entirely.

Minor points:

1. “For first-level GLM analysis, an event of the game presentation had an onset and duration that were the onset timing of the game presentation and 1 s in the Choice phase, respectively. (line 242)” Our understanding is that in this GLM, the critical regressor corresponds to the presentation of the options (i.e., the Choice phase). So the onset of the regressor is set as the onset of this event (if so, it is confusing to refer to this event first as “Game presentation” and then as “Choice phase”). It is unclear what the duration is. What does the “1s in the Choice phase” mean? The first second in the Choice phase? How was the “1s” determined? Why not model the entire Choice phase, or treat the regressor as a transient event (i.e., duration = 0 s)?

2. The authors claimed that they “had a priori hypothesis from previous studies that the amygdala and striatum are involved in inequity”. Their hypothesis regarding inequity aversion and its gender difference does not appear to be in the Introduction. If the authors did have a hypothesis regarding inequity aversion, this need to be stated more clearly. In addition, the authors listed several papers about inequity aversion. How did they determined the coordinates of inequity aversion based on these papers? Simply averaging the coordinates across these papers, or carrying out a coordinate-based meta-analysis? On a related note, the authors did not seem to have “a priori hypothesis” regarding guilt aversion though, which is a bit surprising given that their 2015 paper used exactly the same task.

3. The authors included a section called “Region of interest analysis”. Which analyses are these ROIs for? Why are amygdala and striatum not mentioned here given that the authors “had a priori hypothesis from previous studies that the amygdala and striatum are involved in inequity”? In addition, Baumgartner et al., 2011 cited here is not a meta-analysis paper. Perhaps the authors meant this paper “Bartra et al., 2013, The valuation system: a coordinate-based meta-analysis of BOLD fMRI experiments examining neural correlates of subjective value. Neuroimage”?
