# Supplementary material for: Functional Connectivity Basis and Underlying Cognitive Mechanisms for Gender Differences in Guilt Aversion
Source: eNeuro. 2021 Dec 15;8(6):ENEURO.0226-21.2021. doi: 10.1523/ENEURO.0226-21.2021 (PMC8675089; doi:10.1523/ENEURO.0226-21.2021)
Supplement: Extended Data Figure 1-1 — The actual assignment of x, y, z and τA for the 45 trials. Download Figure 1-1, DOCX file. [file enu-eN-NWR-0226-21-s02.docx]

**Extended Data Figure 1-1. The actual assignment of** $\boldsymbol{x}$**,** $\boldsymbol{y}$**,** $\boldsymbol{z}$ **and**
$\tau_{A}$**for the 45 trials.**

$$\times{10}^{-3}$$

|  | $z_{A}$ | $z_{B}$ | $y_{A}$ | $y_{B}$ | $x_{A}$ | $x_{B}$ | $\tau_{A}$ |
| --- | --- | --- | --- | --- | --- | --- | --- |
| 1 | 600 | 600 | 550 | 890 | 1450 | 690 | 0.8 |
| 2 | 300 | 300 | 220 | 910 | 780 | 650 | 0.8 |
| 3 | 400 | 400 | 320 | 1060 | 840 | 700 | 0.8 |
| 4 | 400 | 200 | 320 | 850 | 880 | 350 | 0.7 |
| 5 | 500 | 200 | 400 | 990 | 780 | 450 | 0.9 |
| 6 | 600 | 200 | 510 | 700 | 700 | 370 | 0.8 |
| 7 | 600 | 600 | 590 | 1150 | 1190 | 900 | 0.9 |
| 8 | 350 | 200 | 320 | 680 | 410 | 350 | 1 |
| 9 | 600 | 200 | 310 | 750 | 1000 | 370 | 0.6 |
| 10 | 700 | 300 | 560 | 1000 | 860 | 550 | 1 |
| 11 | 400 | 200 | 270 | 600 | 860 | 430 | 0.9 |
| 12 | 400 | 400 | 370 | 720 | 920 | 560 | 0.9 |
| 13 | 500 | 500 | 320 | 900 | 880 | 810 | 1 |
| 14 | 800 | 300 | 620 | 810 | 1000 | 500 | 0.8 |
| 15 | 700 | 400 | 600 | 860 | 1100 | 650 | 0.9 |
| 16 | 600 | 100 | 500 | 730 | 670 | 350 | 0.8 |
| 17 | 500 | 500 | 400 | 1000 | 1140 | 800 | 0.7 |
| 18 | 700 | 700 | 510 | 1250 | 1360 | 960 | 0.6 |
| 19 | 800 | 100 | 450 | 680 | 1180 | 440 | 1 |
| 20 | 800 | 500 | 580 | 1270 | 1630 | 680 | 0.6 |
| 21 | 600 | 400 | 400 | 580 | 1180 | 500 | 0.7 |
| 22 | 500 | 500 | 390 | 670 | 860 | 600 | 0.7 |
| 23 | 500 | 200 | 360 | 640 | 680 | 350 | 0.8 |
| 24 | 700 | 400 | 620 | 1250 | 1310 | 650 | 0.9 |
| 25 | 800 | 400 | 660 | 750 | 1160 | 550 | 0.8 |
| 26 | 600 | 100 | 500 | 760 | 890 | 330 | 0.9 |
| 27 | 500 | 500 | 300 | 1080 | 1080 | 950 | 1 |
| 28 | 600 | 600 | 370 | 1000 | 1080 | 890 | 0.6 |
| 29 | 800 | 800 | 550 | 1220 | 1760 | 1020 | 0.6 |
| 30 | 700 | 200 | 530 | 900 | 1180 | 390 | 0.9 |
| 31 | 700 | 600 | 590 | 880 | 1380 | 660 | 0.6 |
| 32 | 700 | 300 | 600 | 970 | 1320 | 570 | 1 |
| 33 | 500 | 200 | 420 | 990 | 530 | 350 | 0.8 |
| 34 | 800 | 400 | 720 | 1250 | 1050 | 550 | 0.7 |
| 35 | 600 | 200 | 390 | 670 | 880 | 350 | 0.8 |
| 36 | 600 | 300 | 260 | 800 | 1080 | 400 | 1 |
| 37 | 700 | 300 | 540 | 740 | 980 | 450 | 1 |
| 38 | 700 | 400 | 530 | 660 | 1130 | 440 | 0.6 |
| 39 | 400 | 400 | 260 | 940 | 880 | 830 | 0.9 |
| 40 | 600 | 400 | 520 | 1220 | 880 | 550 | 0.8 |
| 41 | 500 | 100 | 420 | 1060 | 800 | 180 | 0.8 |
| 42 | 500 | 500 | 460 | 800 | 980 | 540 | 0.9 |
| 43 | 600 | 600 | 470 | 1170 | 930 | 850 | 0.9 |
| 44 | 300 | 200 | 260 | 510 | 430 | 350 | 0.8 |
| 45 | 600 | 200 | 520 | 770 | 780 | 360 | 1 |
