# Supplementary material for: Functional Connectivity Basis and Underlying Cognitive Mechanisms for Gender Differences in Guilt Aversion
Source: eNeuro. 2021 Dec 15;8(6):ENEURO.0226-21.2021. doi: 10.1523/ENEURO.0226-21.2021 (PMC8675089; doi:10.1523/ENEURO.0226-21.2021)
Supplement: Extended Data Figure 1-2 — Instructions for the first and second experiments in the fMRI study. Download Figure 1-2, DOCX file. [file enu-eN-NWR-0226-21-s03.docx]

**Extended Data Figure 1-2. Instructions for the first and second experiments in the fMRI study.**

$$\times{10}^{-3}$$

The following is the instructions provided to the participants.

Instructions for the first experiment

Here we explain the experiment on Day 1. Please read carefully and understand the rules of the experiment.

1. General introduction

In this experiment, you will be paired with another person. In each pair, one person will be player A and the other will be player B. Your role is player A. Player B is a participant of the fMRI experiment on Day 2. Because participants in the fMRI experiment are chosen from among those in the experiment on Day 1, please assume player B participated on Day 1. You and your partner (player B) will be participating in a game that will be explained later. The experiment is strictly anonymous; that is, your identity will not be revealed to others, and others’ identities will not be revealed to you. The earnings paid to you is determined by the decision of you and your partner, as we will explain later.

1. Rules of the game

Please review and ensure that you understand the rules of the game by referring to the following figure. First, player A chooses either "In" or "Out". If player A chooses "Out," player A will get $z_{A}$ yen and player B will get $z_{B}$ yen.

If player A chooses "In", then player B chooses either “Roll” or “Don't Roll” in the Day 2 experiment. If player B chooses "Roll," player A will receive $x_{A}$ yen and player B receives $x_{B}$ yen. If player B chooses " Don't Roll," player A receives $y_{A}$ yen and player B receives $y_{B}$ yen.

1. Procedure of the experiment
2. Look at the "Answer Sheet." $x_{A}$, $x_{B}$, $y_{A}$, $y_{B}$, $z_{A}$, and $z_{B}$ show a specific amount of money. First, check the amount of money. After that, please choose either "In" or "Out" and reveal your belief probability (from 0% to 100% in increments of 10% steps) that you expect your partner (player B) will choose “Roll” based on the amount of money.
3. Please indicate your choices in the "Answer Sheet." Note that the choices made by player A, including you, will be used when player B makes their choice in the fMRI experiment on Experiment Day 2.
4. Payment for you

The choice you make as player A will be used when player B makes their decision in the Day 2 fMRI experiment. However, in this experiment, many participants make decisions as player A, just as you did; therefore, it is uncertain whether the decision you make as player A will be used in the fMRI experiment. If your choice is used in the fMRI experiment, you will receive money according to the choices made by your partner and you. Therefore, if you choose "Out" in today’s experiment and the earnings are already fixed, you will not be paid if it is not used in the fMRI experiment. We will contact you again if we need to pay.

This concludes the explanation of the experiment. Now, please fill in your choices on the "Answer Sheet." You will have 5 minutes to answer. If you have any questions, please raise your hand.

**Player A (You)**

Out

In

**Player B**

**(participants on Day 2)**

Roll

Don’t Roll

**A:** $z_{A}$ **yen**

**B:** $z_{B}$ **yen**

**A:** $y_{A}$ **yen**

**B:** $y_{B}$ **yen**

**A:** $x_{A}$ **yen**

**B:** $x_{B}$ **yen**

Instructions for the second experiment (fMRI experiment)

We will now explain the experiment on Day 2. You will receive 1,200 yen as a participation fee in this experiment. You will receive additional earnings depending on the results of your choices during the experiment as explained below.

1. General introduction

In the Day 2 experiment, you will perform a task in the fMRI scanner. In this experiment, there are two players: player A and player B, and your role is player B. Player A is a participant in the Day 1 experiment other than yourself. In the Day 2 experiment, the decision-making of player A is based on the choices made by participants in the Day 1 experiment.

In each trial, you will be paired with another person who has the role of player A and participate in the game described below. This experiment is repeated 45 times, but your role remains unchanged throughout the 45 trials. A different person is assigned as your partner (player A) by the experimenter each time, so you will not be paired with the same person twice throughout the 45 trials. The experiment is strictly anonymous; that is, your identity will not be revealed to others, and others’ identities will not be revealed to you. The earnings for you are determined by the decision of you and your partner, as we will explain later.

1. Rules of the game

In the Day 1 experiment, player A chooses either "In" or "Out". If player A chooses "Out," player A will get $z_{A}$ yen and player B will get $z_{B}$ yen. If player A chooses "In", then player B chooses either “Roll” or “Don't Roll.” However, player B makes the choice without knowing which option player A has chosen. Therefore, player B should assume that player A has chosen “In.” If player A chooses "In" and player B chooses "Roll," player A will receive $x_{A}$ yen and player B receives $x_{B}$ yen. If player A chooses "In" and player B chooses " Don't Roll," player A receives $y_{A}$ yen and player B receives $y_{B}$ yen. $x_{A}$, $x_{B}$, $y_{A}$, $y_{B}$, $z_{A}$, and $z_{B}$ yen will change with each trial, so be sure to check the amount presented on the computer screen every time.

1. Procedure of the experiment
2. At the beginning of the trial, the experimenter determines your partner (Player A). Your partner will be one of the participants in the Day 1 experiment. However, you will not know who your partner is, and you can only pair with the same person once.
3. Now, look at Screen 1. At the start of the trial, a yellow cross is presented at the center of the screen. Look carefully at the cross while it is displayed. After the yellow cross has changed to green, the screen will switch to the decision-making screen (Screen 2). If the cross turns green, think of it as a signal that the decision-making is about to begin.

[Screen 1]

1. Next, look at Screen 2. You will see a diagram indicating a specific amount of money for each choice and a pie chart representing player A’s belief probability that you will choose “Roll” The belief probability also is based on the choices in the experiment on Day 1. Please check the money and player A’s belief probability on this screen, because both the amount of money and the percentage of belief probability change with each trial. Screen 2 is different for each trial but is presented for 5 s. Player A has already made a decision, but without knowing player A’s choice, you assume that player A has chosen “In” and make your own decision, either “Roll” or “Don’t Roll”, within the time limit. Press the button as quickly and accurately as possible. Please note that the screen will continue to be displayed for 5 s even if you have pressed the button.


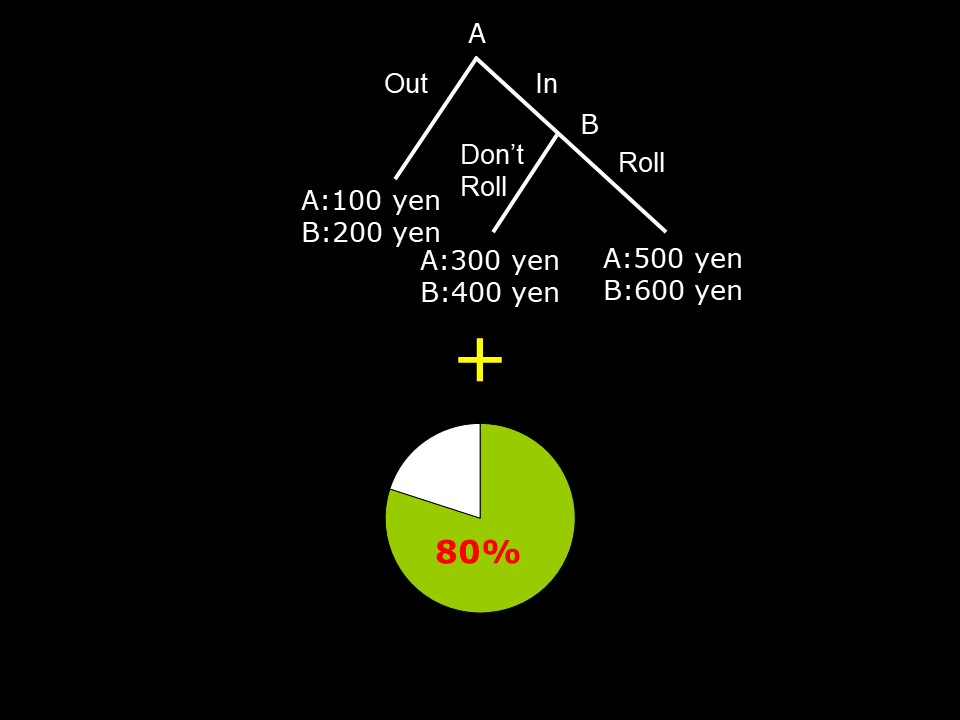


[Screen 2]

1. The second trial will begin after the presentation of Screen 2 is finished. A different partner (player A) will be assigned by the experimenter, and you will perform the same task as described above. The experiment will be terminated after 45 trials of the same task. You will not know the results of each trial until after the experiment is complete.
2. Payment for you

Your earnings will be equal to the amount of money you earned from the 45 decisions you made as player B multiplied by 0.1 plus the participation fee of 1,200 yen. More specifically, the following formula is used:

Your earnings = amount earned from 45 trials × 0.1 (yen) + participation fee of 1,200 yen

Your decisions directly affect yours and your partner’s earnings, so please concentrate on the task during the experiment. In each trial, make sure to check the amount of money and the percentage of belief probability before making your choice. In this experiment, you have a limited time to press the button. Please do not forget to press the button.

This concludes the explanation of the experiment. There will be time for any questions. Afterwards, you will take a confirmatory test. After the explanation of the test, you will practice how to press the button and how to view the screen. After that, you will enter the fMRI scanner and begin the actual experiment.
