# Supplementary material for: Functional Connectivity Basis and Underlying Cognitive Mechanisms for Gender Differences in Guilt Aversion
Source: eNeuro. 2021 Dec 15;8(6):ENEURO.0226-21.2021. doi: 10.1523/ENEURO.0226-21.2021 (PMC8675089; doi:10.1523/ENEURO.0226-21.2021)
Supplement: Extended Data Figure 3-1 — Activities related with Guilt in both genders. Download Figure 3-1, DOCX file. [file enu-eN-NWR-0226-21-s04.docx]

**Extended Data Figure 3-1. Activities related with Guilt in both genders.**

$$\times{10}^{-3}$$

| Brain area | MNI coordinates | | | Voxel size (k) | *t* value |
| --- | --- | --- | --- | --- | --- |
|  | *x* | *y* | *z* |  |  |
| R. DLPFC and DMPFC | 44 | 34 | 26 | 5071 | 9.84 |
| L. DLPFC | -48 | 28 | 34 | 1826 | 10.66 |
| R. Middle Temporal Cortex | 64 | -28 | -16 | 335 | 7.18 |
| R. Parietal Cortex | 44 | -56 | 42 | 2324 | 9.34 |
| L. Parietal Cortex | -44 | -56 | 36 | 1403 | 7.85 |
| R. Occipital Cortex | 18 | -92 | -6 | 6374 | 15.89 |

Notes: MNI coordinates (*x, y*, *z*) indicate the location of the peak correlation. Voxel sizes show the number of supra-threshold voxels, and *t* values correspond with the peak activation voxels. For the whole brain analysis, the threshold was set at *P* < 0.05 FWE corrected. R: right; L: left.
