# Supplementary material for: Functional Connectivity Basis and Underlying Cognitive Mechanisms for Gender Differences in Guilt Aversion
Source: eNeuro. 2021 Dec 15;8(6):ENEURO.0226-21.2021. doi: 10.1523/ENEURO.0226-21.2021 (PMC8675089; doi:10.1523/ENEURO.0226-21.2021)
Supplement: Extended Data Figure 3-2 — Activities related with Inequity in both genders. Download Figure 3-2, DOCX file. [file enu-eN-NWR-0226-21-s05.docx]

**Extended Data Figure 3-2. Activities related with Inequity in both genders.**

| Brain area | MNI coordinates | | | Voxel size (k) | *t* value |
| --- | --- | --- | --- | --- | --- |
|  | *x* | *y* | *z* |  |  |
| R. Ventral Striatum | 10 | 8 | -2 | 67 | 4.06 |
| L. Ventral Striatum | -10 | 8 | 2 | 105 | 4.31 |
| R. DLPFC and DMPFC | 32 | 26 | 46 | 2774 | 4.77 |
| R. VLPFC | 50 | 46 | 2 | 210 | 4.14 |
| L. VLPFC | -50 | 18 | -6 | 110 | 3.94 |
| R. Inferior Temporal Cortex | 54 | -52 | -18 | 729 | 5.23 |
| L. Pre-supplementary Motor Area | -46 | 0 | 50 | 263 | 4.57 |
| Middle Cingulate Cortex | -2 | -28 | 26 | 1075 | 4.62 |
| L. Inferior Temporal Cortex | -60 | -32 | -14 | 551 | 5.21 |
| R. Parietal Cortex | 30 | -70 | 56 | 157 | 4.12 |
|  | 44 | -48 | 56 | 1258 | 4.49 |
|  | 10 | -70 | 18 | 116 | 4.00 |
| L. Parietal Cortex | -48 | -54 | 28 | 965 | 4.24 |
|  | -14 | -70 | 20 | 169 | 4.43 |
|  | -2 | -70 | 60 | 180 | 3.96 |
| R. Occipital Cortex | 34 | -78 | -16 | 487 | 4.71 |

Notes: MNI coordinates (*x, y*, *z*) indicate the location of the peak correlation. Voxel sizes show the number of supra-threshold voxels, and *t* values correspond with the peak activation voxels. For the whole brain analysis, the threshold was set at *P* < 0.001 uncorrected; activity in the bilateral ventral striatum was maintained after small volume FWE corrections at *P* < 0.05. R: right; L: left.
