# Supplementary material for: Functional Connectivity Basis and Underlying Cognitive Mechanisms for Gender Differences in Guilt Aversion
Source: eNeuro. 2021 Dec 15;8(6):ENEURO.0226-21.2021. doi: 10.1523/ENEURO.0226-21.2021 (PMC8675089; doi:10.1523/ENEURO.0226-21.2021)
Supplement: Extended Data Figure 3-5 — Results of the PPI analysis for VMPFC × Inequity in both genders. Download Figure 3-5, DOCX file. [file enu-eN-NWR-0226-21-s08.docx]

**Extended Data Figure 3-5. Results of the PPI analysis for VMPFC × Inequity in both genders.**

| Brain area | MNI coordinates | | | Voxel size (k) | *t* value |
| --- | --- | --- | --- | --- | --- |
|  | *x* | *y* | *z* |  |  |
| L. Striatum | -2 | 14 | 2 | 7 | 3.58 |
| L. Orbitofrontal Cortex | -30 | 46 | -4 | 4 | 3.41 |

Notes: MNI coordinates (*x, y*, *z*) indicate the location of the peak correlation. Voxel sizes show the number of supra-threshold voxels, and *t* values correspond with the peak activation voxels. For the whole brain analysis, the threshold was set at *P* < 0.001 uncorrected. L: left.
