# Supplementary material for: Functional Connectivity Basis and Underlying Cognitive Mechanisms for Gender Differences in Guilt Aversion
Source: eNeuro. 2021 Dec 15;8(6):ENEURO.0226-21.2021. doi: 10.1523/ENEURO.0226-21.2021 (PMC8675089; doi:10.1523/ENEURO.0226-21.2021)
Supplement: Extended Data Figure 4-1 — Differences of activities related to guilt between men and women. Download Figure 4-1, DOCX file. [file enu-eN-NWR-0226-21-s09.docx]

**Extended Data Figure 4-1. Differences of activities related to guilt between men and women.**

$$\times{10}^{-3}$$

| Brain area | MNI coordinates | | | Voxel size (k) | *t* value |
| --- | --- | --- | --- | --- | --- |
|  | *x* | *y* | *z* |  |  |
| R. VMPFC | 10 | 42 | -16 | 37 | 3.74 |
| R. Parietal Cortex | 54 | -58 | 48 | 20 | 3.64 |

Notes: MNI coordinates (*x, y*, *z*) indicate the location of the peak correlation. Voxel sizes show the number of supra-threshold voxels, and *t* values correspond with the peak activation voxels. For the whole brain analysis, the threshold was set at *P* < 0.001 uncorrected; activity in the right VMPFC was maintained after small volume FWE corrections at *P* < 0.05. R: right.
