# Supplementary material for: Functional Connectivity Basis and Underlying Cognitive Mechanisms for Gender Differences in Guilt Aversion
Source: eNeuro. 2021 Dec 15;8(6):ENEURO.0226-21.2021. doi: 10.1523/ENEURO.0226-21.2021 (PMC8675089; doi:10.1523/ENEURO.0226-21.2021)
Supplement: Extended Data Figure 4-2 — Results of the PPI analysis for guilt when testing for gender differences. Download Figure 4-2, DOCX file. [file enu-eN-NWR-0226-21-s10.docx]

**Extended Data Figure 4-2. Results of the PPI analysis for guilt when testing for gender differences.**

| Brain area | MNI coordinates | | | Voxel size (k) | *t* value |
| --- | --- | --- | --- | --- | --- |
|  | *x* | *y* | *z* |  |  |
| R. DLPFC | 36 | 20 | 26 | 428 | 4.80 |
|  | 50 | 38 | 20 | 42 | 3.90 |
| L. Occipital Cortex | -30 | -88 | -8 | 31 | 3.56 |

Notes: MNI coordinates (*x, y*, *z*) indicate the location of the peak correlation. Voxel sizes show the number of supra-threshold voxels, and *t* values correspond with the peak activation voxels. For the whole brain analysis, the threshold was set at uncorrected *P* < 0.001. R: right; L: left.
